# Discovery of Spiro[chromane-2,4′-piperidine] Derivatives as Irreversible Inhibitors of SARS-CoV‑2 Papain-like Protease

**DOI:** 10.1021/acs.jmedchem.5c03704

**Published:** 2026-02-02

**Authors:** Qiangqiang Wei, Ashley J. Taylor, Nagaraju Miriyala, Mahesh A. Barmade, Zachary O. Gentry, Jordan Anderson-Daniels, Kevin B. Teuscher, Mackenzie M. Crow, Chideraa Apakama, Taylor M. South, Tyson A. Rietz, Kangsa Amporndanai, Jason Phan, John L. Sensintaffar, Mark Denison, Taekyu Lee, Stephen W. Fesik

**Affiliations:** 1 Department of Biochemistry, 12327 Vanderbilt University School of Medicine, Nashville, Tennessee 37232-0146, United States; 2 Department of Pathology, Microbiology, and Immunology, Vanderbilt University Medical Center, Nashville, Tennessee 37232, United States; 3 Department of Pharmacology, 12327 Vanderbilt University School of Medicine, Nashville, Tennessee 37232-6600, United States; 4 Department of Chemistry, 5718 Vanderbilt University, Nashville, Tennessee 37235, United States

## Abstract

The papain-like protease (PL^Pro^) plays a key role in SARS-CoV-2 replication and represents a promising target for the development of new antiviral therapies. Previous efforts to develop fragment-derived inhibitors of PL^Pro^ led to the identification of a novel class of spiro­[chromane-2,4′-piperidin]-4-one inhibitors exemplified by lead compound **7**. High-resolution covalent cocrystal structures and molecular dynamics simulations were utilized to guide the development of a series of low-nanomolar irreversible PL^Pro^ inhibitors, with lead compound **45** demonstrating strong enzymatic inhibition (IC_50_ = 0.059 μM at *T* = 60 min) and antiviral activity in A549 cells (EC_50_ = 2.1 μM at 48 hpi). This novel class of inhibitors represents a promising avenue for the development of therapeutics to overcome the potential of drug-resistant viral strains and future coronavirus outbreaks.

## Graphical abstract

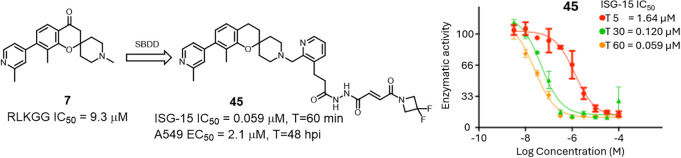

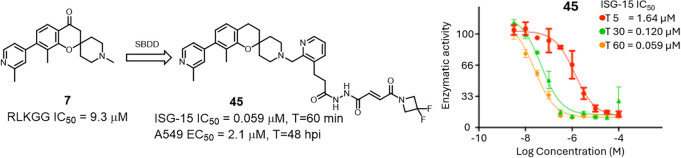

## Introduction

Severe acute respiratory syndrome coronavirus 2 (SARS-CoV-2) was responsible for the COVID-19 pandemic of 2019–2022, which resulted in more than 7 million deaths worldwide.^1,2^ The continual emergence of new viral strains with increased infectivity and resistance to existing treatments remains a global concern.^3,4^ There are currently 3 FDA-approved drugs for treatment of COVID-19, the RNA-dependent RNA polymerase inhibitors molnupiravir (Lagevrio)^5^ and remdesivir (Veklury)^6^ and the main protease inhibitor nirmatrelvir (Paxlovid).^7^ Although these drugs have proven effective in the treatment of COVID-19, the emergence of drug-resistant variants has significantly limited their efficacy.^4,8^ This highlights the need to develop additional anticoronaviral therapies preferably with a different mechanism of action which can be used to develop highly effective single agent therapeutics and combination drug therapies.

SARS-CoV-2 papain-like protease (PL^Pro^) is a cysteine protease that plays a key role in the viral replication cycle through the cleavage of nonstructural proteins 1–3.^9–11^ PL^Pro^ also disrupts the host immune responses by cleaving ubiquitin and interferon-stimulated gene 15 (ISG-15),^12–14^ making it an attractive target for the development of antiviral drugs with a novel mechanism of action. The high homology of PL^Pro^ across the coronaviral family also presents an opportunity for SARS-CoV-2 PL^Pro^ inhibitors to be used as an effective treatment in the future against potential coronavirus outbreaks. Despite the key role PL^Pro^ plays in the viral life cycle, there are currently no inhibitors approved by the FDA or in clinical trials.

PL^Pro^ has a rather unique and specific substrate recognition sequence of LXGG (X = Arg, Lys and Asn),^15–17^ resulting in the S1 & S2 subsites forming a narrow glycine channel which prohibits small molecule binding. As a result, inhibitors are required to bind to the largely solvent exposed S3 & S4 subsites. The highly flexible nature of the BL2 loop^18,19^ which constitutes one side of the active site further complicates inhibitor identification and development. In recent years, several drug discovery campaigns against SARS-CoV-2 PL^Pro^ have been reported, most of which are derived from the original SARS-CoV-1 PL^Pro^ inhibitor GRL-0617 (Figure. [Fig fig1]).^20^ Due to their high homology (83%), GRL-0617 also exhibits inhibitory activity against SARS-CoV-2 PL^Pro^ with an IC_50_ of 2.1 μM.^21^ Reversible inhibitors developed at the University of Illinois and Rutgers University extended into the BL2-groove and S3 subsite improving ligand binding (**1** & **2**);^22,23^ however, weak cellular activity required high doses (200–500 mg/kg BID) to see a pharmacological effect. More potent GRL-0617 analogues developed by Pfizer (**3**)^24^ exhibited activity *in vivo*, which successfully validated PL^Pro^ as an anticoronaviral target.

**Figure 1:**
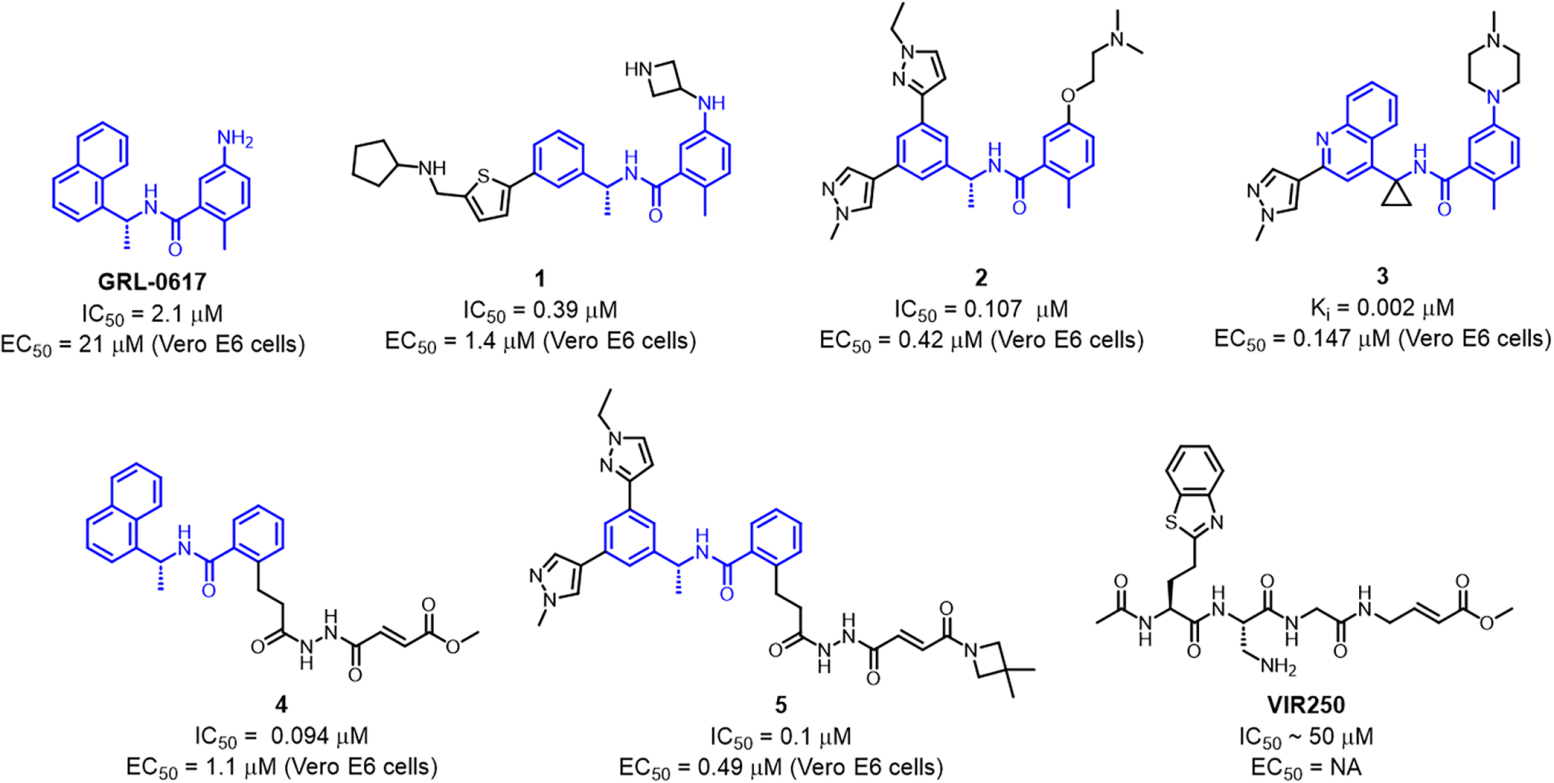
Structures of the first reported PL^Pro^ inhibitor GRL-0617 and its analogues developed by University of Illinois **1** ^23^, Rutgers University **2** & **5** ^22,26^ , Pfizer **3**,^24^ and Oakridge National Laboratory **4** ^25^ with their inhibitory and cellular activity reported. Structure of peptide based irreversible inhibitor **VIR250** developed by the NIH.^27^

Oakridge national laboratory found that inclusion of a *N*,*N*’-diacetylhydrazine peptidomimetic linker on the phenyl ring of GRL-0617 was able to protrude into the glycine channel allowing the development of irreversible inhibitors of PL^Pro^ (compound **4**). Despite developing several nanomolar inhibitors, high metabolic clearance limited their effectiveness in cellular efficacy assays.^25^ To address these concerns, Rutgers University modified the fumarate warhead to further optimize the potency and stability of the GRL-0617 core (compound **5**). However, extensive studies failed to identify compounds with oral bioavailability.^26^ The NIH has also investigated the development of peptide based irreversible inhibitors identifying several low micromolar molecules, but the project did not progress further.^27^ Although previous efforts have fallen short of identifying a clinical lead, PL^Pro^ remains a valid and unaddressed therapeutic target for the treatment of SARS-CoV-2.

Here we describe the development of irreversible PL^Pro^ inhibitors using spiro­[chromane-2,4’-piperidin]-4-one as a core unit. The first high resolution crystal structure of covalently labeled PL^Pro^ shows key binding interactions in the glycine channel and oxyanion hole allowing for the structure-guided development of irreversible inhibitors. This work led to the discovery of a novel class of irreversible PL^Pro^ inhibitors that exhibit low nanomolar time-dependent inhibition in an enzymatic assay.

## Results and Discussion

### Initial Irreversible Inhibitor Design and Synthesis

Previously, we have described an NMR fragment-based screen of PL^Pro^, and identified a novel spiro­[chromane-2,4’-piperidin]-4-one scaffold that binds to PL^Pro^ (**6**, IC_50_ = 340 μM).^28^ Subsequent structure–activity relationship (SAR) exploration around the BL2-groove led to compound **7** with an IC_50_ of 9.3 μM, providing an attractive scaffold for the further development of irreversible inhibitors.^29^ A crystal structure of **7** in complex with PL^Pro^ revealed that the core unit occupies the S4 subsite forming π stacking with Tyr-268, while the *N*-methyl piperidinium forms a hydrogen bond with nearby Asp-164 (Figure 2B). The piperidinyl nitrogen was deemed as a suitable vector for expansion into the S3 subsite allowing for access through the glycine channel to the active Cys-111 (Figure 2A), which is positioned ∼ 8.5 Å away from the *N*-Me of **7.**

**Figure 2:**
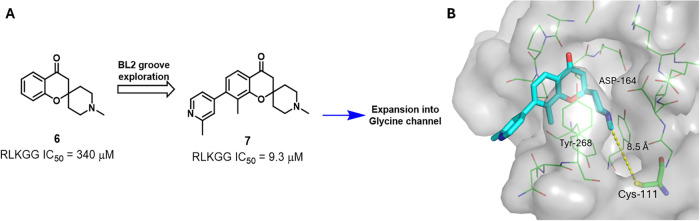
(A) Design strategy of irreversible PL^Pro^ inhibitors. (B) X-ray crystal structure of PL^Pro^ in complex with **7** (teal sticks, PDB 9Z0C), the yellow dash indicates the distance between methylpiperidine and Cys-111.

To assess the feasibility of covalent modification of Cys-111, a series of 25 inhibitors containing flexible linkers of varying length and an array of conventional Michael acceptors that can react with cysteine were prepared and tested (Figure 3). A biochemical inhibition assay using a fluorescently labeled polypeptide (Ac-RLKGG-AMC) to mimic the natural substrate was developed. The IC_50_ was determined 10 min after the substrate addition, which is a required minimum time point to accumulate a sufficient level of fluorescent signal due to the weak binding affinity of the substrate, to assess the initial binding affinity. In addition, a degree of time dependent inhibition was determined by measuring IC_50_ after a 60 min incubation and comparing with the initial binding affinity. Of the compounds tested only the fumarate ester and *N*,*N*’-diacetylhydrazine linker showed any sign of inhibitory activity but failed to show time dependent inhibition. Based on these initial results, a second series of inhibitors focusing on the methyl fumarate warhead was devised using a flexible carbon chain to determine the ideal length and conformation required to access the catalytic site (Table 1). Compared to compound **7**, a linker length of atoms 3 or greater was found to improve compound binding with the 4-atom linker proving to be the optimal yielding a 20-fold increase in potency (compound **10**, IC_50_ = 0.5 μM). Both the 1 and 2 atom linkers failed to bind, suggesting the warhead failed to access the glycine channel. Although compounds **10** and **11** displayed improved inhibitory activity, there was no sign of time dependent inhibition. It was concluded that covalent labeling was not observed for one of two reasons. One possibility was that the highly flexible linker could limit access to the active site with the warhead instead projecting into the top of the S3 pocket. Alternatively, the fumarate warhead may not be correctly positioned in the glycine channel and is unable to react with Cys-111. The fact that covalent modification of the catalytic cysteine was not impacted by linker length suggested that failure to access the glycine channel was the primary reason for lack of time dependent inhibition.

**Figure 3:**
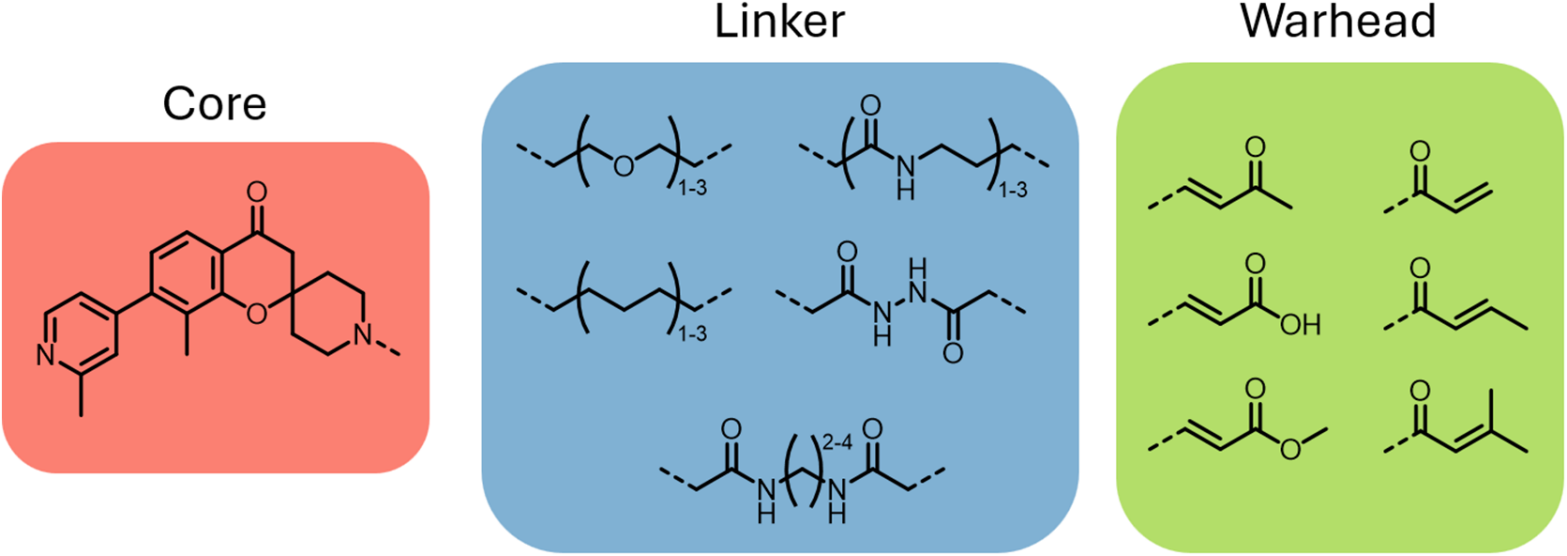
Initial attempts to develop irreversible inhibitors of PL^Pro^ with elaborated fragment hit (red) and several tested linkers (blue) and Michael acceptors (green) combinations shown.

**Table 1:**
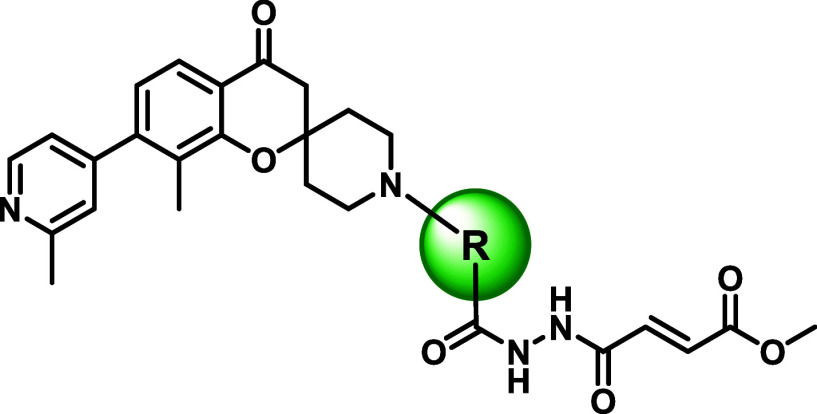

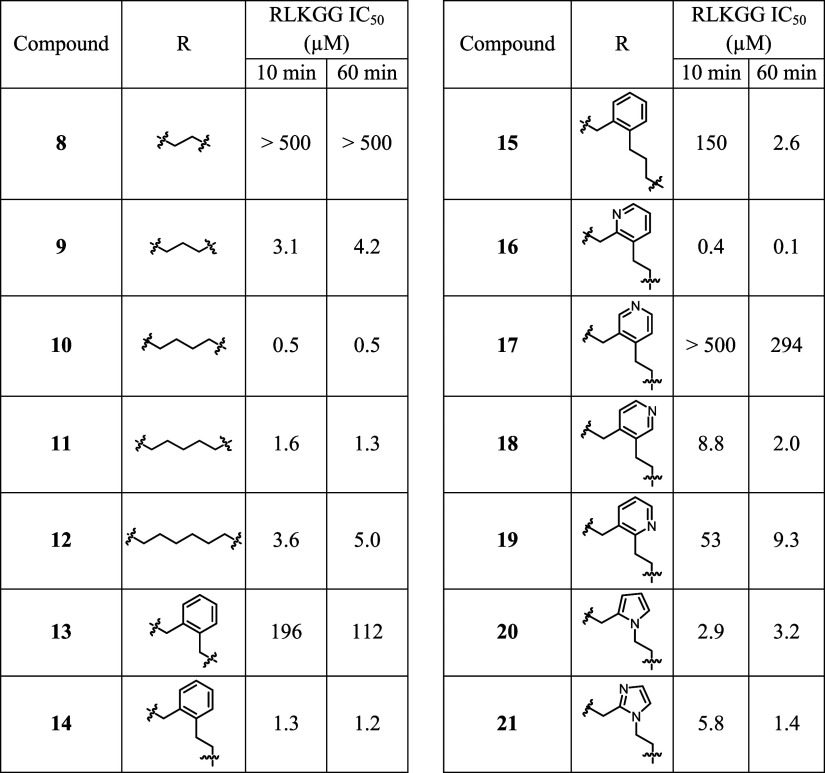
Enzyme Inhibition Data for Covalent Linkers **8**–**21** ^a^

a RLKGG biochemical IC_50_ represents the average of a minimum of two replicates.

It was hypothesized that adding rigidity to the linker would restrict the conformational rotation to provide a more stable vector for the fumarate ester to access the glycine channel, further improving inhibitory activity and the rate of covalent modification. Previously reported inhibitors (**1** – **5**) showed that inclusion of a phenyl in the S3 subsite could improve binding and offered a suitable vector for expansion into the glycine channel. However, the different binding position of our spiro­[chromane-2,4’-piperidin]-4-one core and presence of a basic nitrogen raised concerns over the suitability of a phenyl linker. Molecular dynamics simulations were conducted to assess conformational dynamics of several heteroaryl linker units on our tricyclic core in aqueous solution. Two key dihedral angles (piperidine nitrogen to methylene linker and methylene linker to heterocycle) were measured and compared with the predicted optimal binding pose required to access the glycine channel based on X-ray crystal structures and modeling (black arrow in Figure 4). Simulations were run for 100 ns with snapshots taken every 0.02 ns for a total of 5002 frames, the dihedral angles were recorded and used to generate contour maps of adopted conformations. It was found that an unsubstituted phenyl exhibited a large degree of conformational flexibility adopting 5 different poses in solution (Figure 4B), but none of them offered a suitable vector for expansion into the glycine channel. Simulations with the 2-pyridyl analogue revealed that the protonated piperidine nitrogen could form an intramolecular hydrogen bond with the pyridine nitrogen greatly restricting conformational flexibility of the linker unit in solution (Figure 4C). Moreover, the dihedral angles of the two observed conformers were notably different from all other simulated heterocycles with the major pose (47% of conformers) overlapping more closely with the predicted ideal binding orientation. Interestingly, introduction of a nitrogen in the 3-position had the opposite effect with electrostatic repulsions causing a greater degree of conformational flexibility resulting in a dihedral dispersion profile similar to the phenyl linker with 6 major conformations being observed (Figure 4D). MD simulations also suggested that 5-membered heterocyclic linkers could adopt the desired binding orientation, but they did not provide an ideal exit vector to access the glycine channel and would be predicted to negatively affect binding.

**Figure 4:**
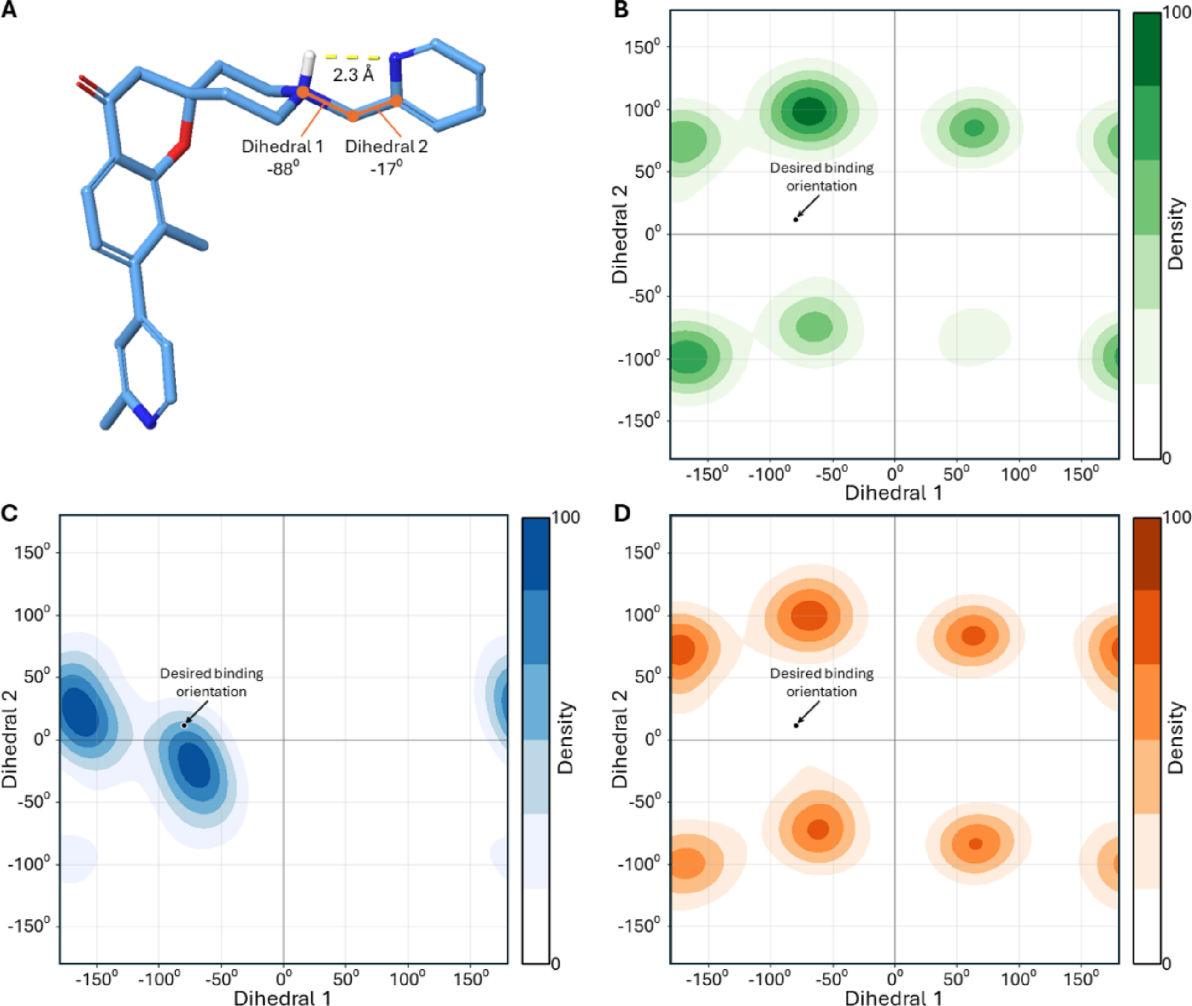
(A) Most commonly adopted pose of 2-pyridine analogue in aqueous solution shown as blue sticks with the two key dihedral angles measured in orange. (B-D) Contour maps of dihedral angle distribution for phenyl, 2-pyridyl and 3-pyridyl analogues in solution colored green, blue and orange respectively with predicted optimal binding pose required to access the glycine channel shown as a black arrow.

An initial series of phenyl linkers (compounds **13**-**15**) found that a two-carbon spacer was essential for activity with the 1 and 3-carbon spacers being 150 and 100-fold less potent, respectively. Although phenyl linker **14** maintained binding, there was no improvement in its inhibitory activity, and we once again failed to see any sign of covalent modification at the 60 min time point. Inclusion of a nitrogen at the 2-position of phenyl **14** improved initial binding 3-fold, supporting our predictions about reduced conformational flexibility being beneficial to binding. Indeed, compound **16** exhibited the highest inhibitory activity among compounds in Table 1 and was the first tested analogue which showed time dependent inhibition of PL^Pro^ with a 4-fold improvement in inhibition between the 10 and 60 min time points (IC_50_ = 0.4 and 0.1 μM respectively). Surprisingly, the predicted negative impact of the 3-pyridyl’s electrostatic repulsion was more pronounced than expected with a 3000-fold decrease in potency between compounds **16** and **17**. The inclusion of a 4 or 5-pyridyl likewise failed to improve binding, but their negative effect was less severe. It is worth noting that all pyridyl analogues exhibited time dependent inhibition of PL^Pro^ with a ∼ 5-fold improvement in IC_50_’s at the 60 min time point regardless of their initial binding affinities. As predicted, incorporation of 5 membered heterocycles such as pyrrole and imidazole (**20** and **21**) maintained binding but were not as potent as their 6 membered phenyl and 2-pyridyl analogues. Interestingly, both compounds showed similar potency at the 10 min time point, but moderate time dependent inhibition was only observed for imidazole **21**. These results highlight the importance of the 2- pyridyl linker to correctly orient the warhead for access to the glycine channel and to initiate the covalent modification of Cys-111.

### Optimization of the Glycine Peptidomimetic and Fumarate Warhead

The *N*,*N*’-diacetylhydrazine and fumarate methyl ester warhead were introduced to accommodate the narrow glycine channel and facilitate the covalent binding with Cys-111. Previously published compounds containing these motifs exhibited poor pharmacokinetic profiles in mice characterized by extremely high IV clearance that exceeds the liver blood flow rate and poor oral bioavailability.^25,26^ A series of analogues were synthesized to investigate the contribution of the hydrazine linker and warhead to the overall biological activity and their amenability to modification (Table 2). In short, removal or substitution of either amide in the hydrazine linker resulted in complete loss of binding with no signs of inhibition at the highest tested concentration, highlighting the importance of a glycine-peptidomimetic to access the glycine channel. Attempts to truncate the fumarate ester to either the acrylate or ketone were similarly unsuccessful with a total loss of activity. The ester also played a key role in activity with carboxylic acid analogue **26** being more than 100-fold less active than the methyl ester. It is worth noting that unlike most other modifications, covalent labeling was still observed by **26**, suggesting that the modification of the ester could impact binding affinity rather than warhead reactivity. Extension of the methyl ester to an ethyl or longer carbon chain maintained compound inhibition and warhead reactivity. These results suggested that the ester may be further elaborated to improve activity.

**Table 2:**
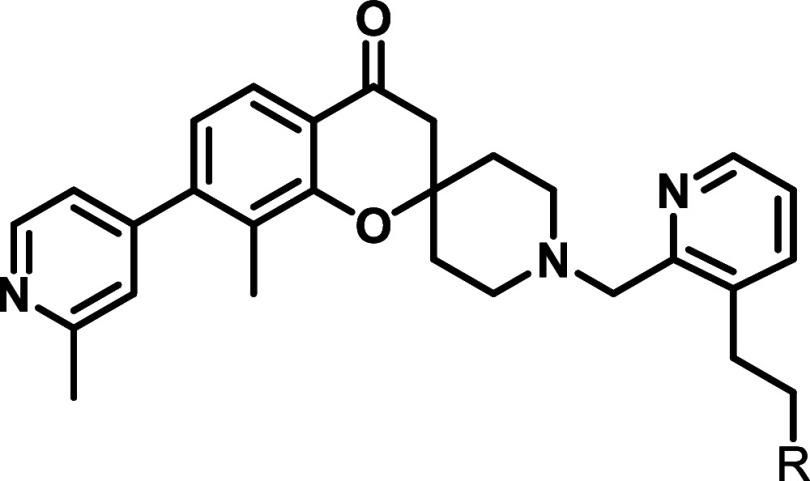

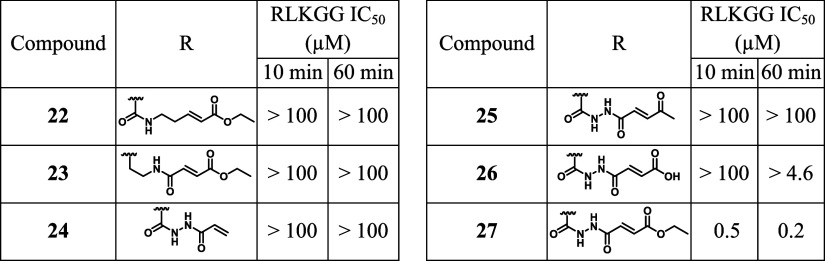
Enzyme Inhibition Data for Covalent Linkers **22**–**27** ^a^

a RLKGG biochemical IC50 and represents the average of a minimum of two replicates.

### Development of an Acrylamide Warhead

Initial testing of **16** and several other fumarate esters in SARS-CoV-2 infected A549 lung cells showed no sign of antiviral activity. Further investigation revealed that the fumarate ester **16** completely hydrolyzed to the significantly less reactive free acid when incubated in RPMI media supplemented with 10% FBS for 12 h at 37 °C. It was proposed that conversion to the more stable acrylamide may improve cellular activity while still providing a synthetic handle to access the oxyanion hole to further enhance ligand binding. Initial profiling of the methyl and dimethyl amides (**28** & **29**) revealed a similar reduction in binding as seen with the free acid analogues; however, warhead reactivity was maintained with a significant increase in IC_50_ between the 10 and 60 min time points. Testing of over 30 substituted amides revealed a subset of cyclized tertiary amides were able to access the oxyanion hole improving ligand binding and warhead reactivity. Table 3 depicts selected examples that showed improved binding affinity with robust time dependent inhibition. Introduction of a morpholine or piperazine ring (**30** & **31**) yielded a > 15-fold increase in binding with low micromolar inhibition observed after 10 min incubation (IC_50_ = 7.0 and 4.1 μM respectively). Removal of hydrogen bond donors through the substitution of the piperazine nitrogen with *i*-propyl **32** enhanced binding affinity with a 4-fold increase in inhibition at the 10 min time point compared to **31** but had no effect at later time points. Further rigidification of the cyclic amides to the smaller azetidine and azaspiro[3.3]­heptane rings improved inhibition with the morpholine and piperazine isosteres **35** & **36** being ∼ 4 times more potent than their 6-membered ring counterparts. A similar trend was observed when fused aromatic rings were linked to the cyclic amide (compounds **37** & **38**). It is also noteworthy that compounds **33**–**38** exhibited high potencies at the 60 minute time point beyond the lower detection limit of our RLKGG enzymatic assay (∼0.1 μM), which required a high concentration of substrate due to its low affinity to PL^Pro^.

**Table 3:**
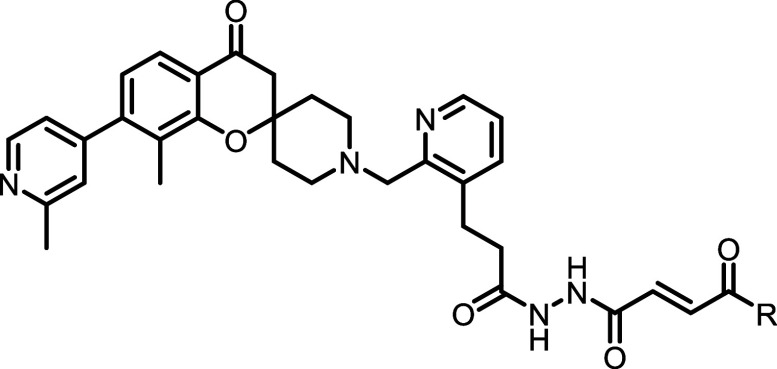

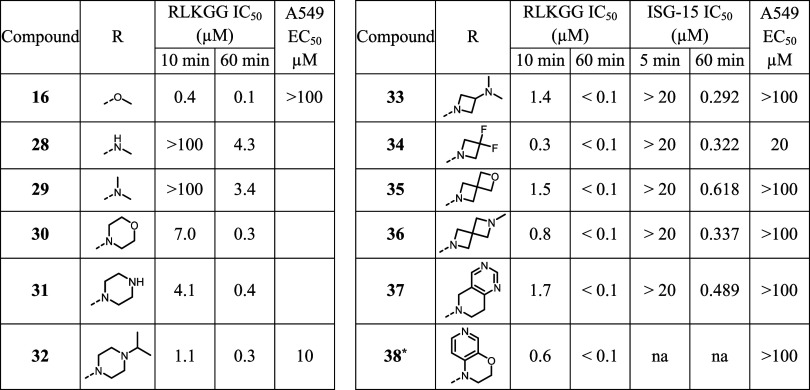
Enzyme Inhibition and Cell Activity Data for Acrylamides **28**–**38** ^a^

a RLKGG biochemical IC_50_ and A549 cellular EC_50_ represents the average of a minimum of two replicates.

^*^Compound **38** showed signs of degradation upon retesting in ISG-15 assay.

As our inhibitors became more potent, it was also necessary to determine IC_50_’s at an earlier time point to minimize the covalent modification of Cys-111 before the first measurement. Therefore, a second enzymatic inhibition assay was developed using fluorescently labeled ISG-15-AMC substrate (K_m_ = 1.564 μM and k_cat_ = 0.024 1/s), allowing us to accurately measure compound IC_50_ and K_i_ to 2 nM. Additionally, the higher sensitivity of this new assay enabled us to characterize compound binding with a less than 5 min incubation allowing for a more accurate differentiation between ligand binding affinity and warhead reactivity. Initial profiling of compounds **33**-**37** under the new assay conditions showed less than 50% inhibition at 20 μM with a 5 min incubation. Unlike the first assay, all compounds exhibited differentiated IC_50_’s ranging from 0.3 to 0.6 μM at the 60 min time point. It is interesting to note that the substituted azetidines **33** & **34** were found to be slightly more active than larger spiro cyclic analogues **35** & **36**. These results suggested that measured >3-fold higher IC_50_’s at 60 min in the new protocol could be due to a stiffer competition with a higher affinity substrate, and inhibitory activities of **33**-**37** were predominated by warhead reactivity. Encouragingly, several acrylamides were beginning to show signs of cellular antiviral activity in A549 cells at higher concentrations with **32** and **34** having an EC_50_ of ∼ 10, and 20 μM at 48 hours post infection (hpi). Although substitution of the fumarate ester to the cyclic acrylamides improved stability and on-target potency, further optimization of cellular antiviral activity was required. It was previously observed that removal of the carbonyl in the spiro chromanone core improved compound permeability and may enable us to further improve cellular activity.

### Optimization of Spiro Chromane Acrylamides

A series of acrylamides were synthesized using the spiro chromane core with a focus on spirocyclic and fluoro substituted azetidine amides (compounds **39**-**50**), and their inhibitory activities were profiled using the ISG-15 assay. Removal of the carbonyl in the core was found to be highly beneficial to ligand binding with all compounds exhibiting significantly enhanced binding affinity compared to their corresponding chromanone analogues with IC_50_’s ranging from 1.6 to 9.0 μM at the 5 min time point (Table 4). Strong time dependent inhibition was also observed with a 30–60-fold increase in IC_50_ between the 5 and 60 min time points. Similar SAR trends were observed as with the chromanone core with the more rigid azetidine and spirocycles being twice as active as the flexible cyclohexyl analogues. Inclusion of electron withdrawing groups further improved inhibition with difluoro and cyano azetidine (compounds **45** & **50**) proving to be the most potent with a IC_50_ of 59 and 40 nM after a 60 min incubation. As hypothesized, removal of the carbonyl was also beneficial to cellular activity with all tested compounds showing signs of antiviral activity in A549 cells, additionally all tested compounds showed no signs of cytotoxicity at the highest concentrations of 100 μM. Compound **45** was found to have the highest antiviral activity with a cellular EC_50_ of 2.1 μM at 48 hpi. Despite having a similar IC_50_’s, the addition of a cyano group was found to negatively impact cellular activity with compound **50** having the lowest EC_50_ of all compounds tested. Profiling of several spiro chromanone and chromane compounds revealed the cyclic acrylamides exhibited good kinetic solubility (97–139 μM) and moderate microsomal stability (t_1/2_ 22–71 min). However, most compounds had poor PAMPA permeability (logP_app_ < −6.2), which may be the major contributing factor for the lower levels of cellular activity and lack of correlation between IC_50_ and EC_50_. Previous work detailing optimization of the BL2 groove noted its tolerance for a large variety of heterocycles and the potential to use these substitutions to optimize the pharmacokinetic properties of future compounds.^29^ This strategy could be employed in the future to improve the permeability of our lead compounds and further increase cellular activity.

**Table 4:**
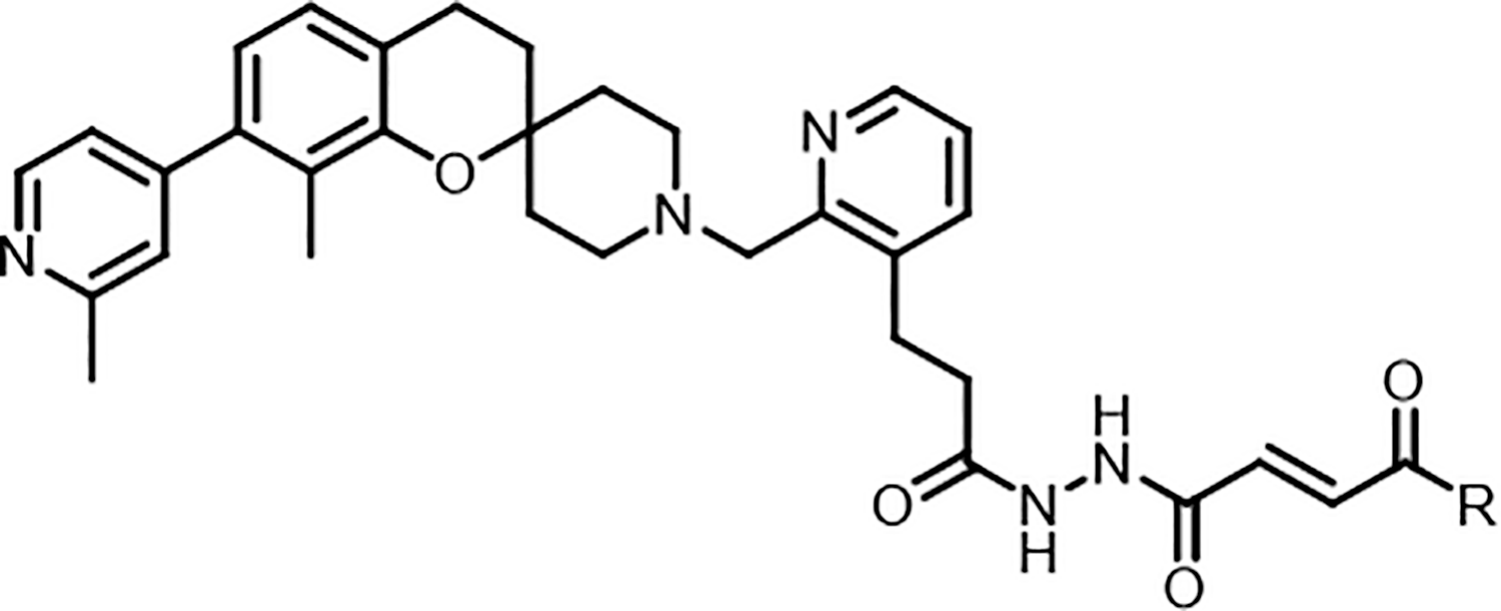

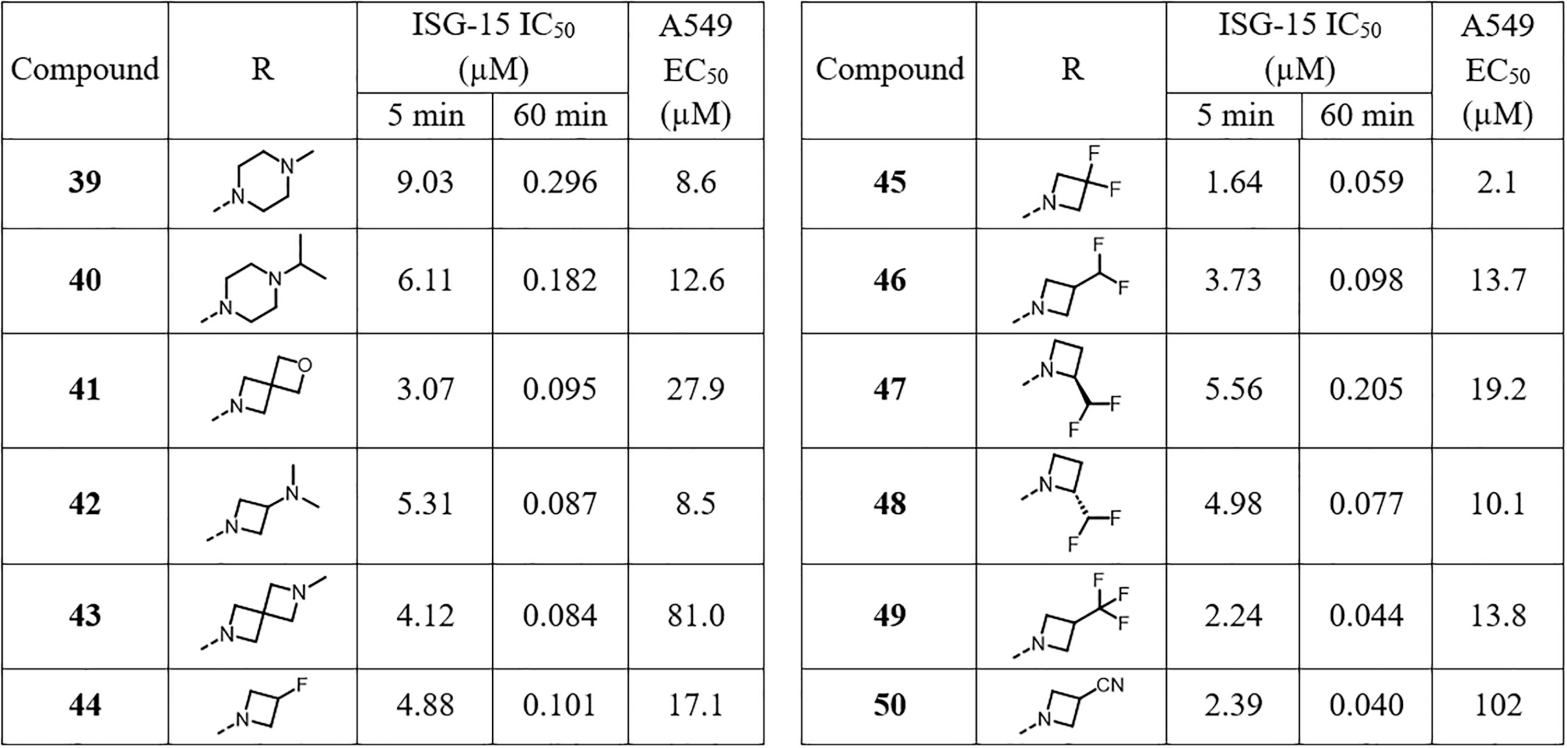
Enzyme Inhibition and Cell Activity Data for Des-carbonyl Acrylamides **39**–**50** a

a ISG-15 biochemical IC_50_ and A549 cellular EC_50_ represents the average of a minimum of two replicates.

The improved potency and stability of the cyclic acrylamides allowed for the development of a covalent cocrystallization procedure which generated a 1.65 Å X-ray structure of compound **41** complexed with PL^Pro^ (Figure 5). This is the first sub 2.5 Å crystal structure of PL^Pro^ covalently bound to a small molecule, allowing for a greater understanding of the binding interactions and the key role played by the *N*,*N*’-diacetylhydrazine linker in accessing the glycine channel. Electron density for the ligand was observed in all 4 subunits and the thioether covalent bond was present and could be accurately modeled in all copies. The spiro chromane core was found to bind higher in the S4 subsite than the reversible inhibitor **7**; however, due to flexibility of the BL2 loop key π-stacking and hydrogen bonding with residues Tyr-268 and Asp-164 were maintained. As predicted by our molecular dynamics simulations, the 2-pyridine sits higher in the S3 pocket and rotates 35 degrees to allow for expansion into the glycine channel while maintaining an intramolecular hydrogen bond with the piperidine nitrogen of the core (Figure 5C). An additional water mediated hydrogen bond with Glu-167 was also observed. The hydrazine linker was found to form a network of 4 hydrogen bonds with the C and N terminals of Gly-163 and Gly-271 explaining why the peptidomimetic linker was essential for binding and was highly resistant to modification. The oxoazaspiro[3.3]­heptane ring sits in the largely solvent exposed S1’ subsite but does not form any notable interactions with nearby residues. However, our SAR results clearly showed that the binding affinity of irreversible inhibitors can be further optimized by introduction of a suitable P1’ moiety. The new structural information may aid the design of analogues targeting additional favorable hydrophobic and electrostatic interactions to further improve compound potency and pharmaceutical properties. In any case, the chromane acrylamides represent a promising class of irreversible PL^Pro^ inhibitors that could lead to the development of clinically useful SARS-CoV-2 therapeutics.

**Figure 5:**
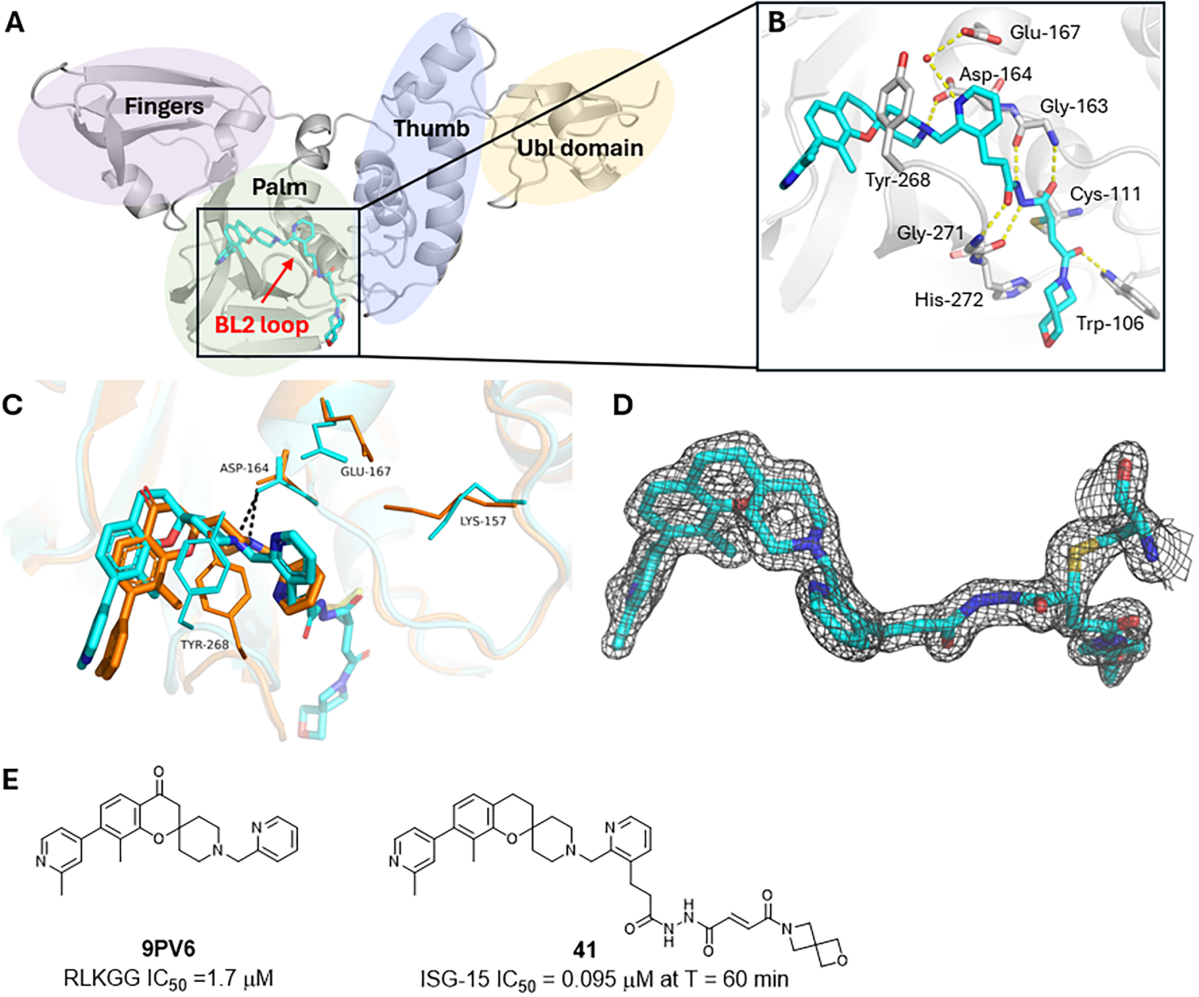
(A) Co-crystal structure of SARS-CoV-2 PL^Pro^ in complex with covalent inhibitor **41** with key protein domains highlighted. (B) PL^Pro^ binding pocket with key interactions between nearby amino acids (gray lines) and **41** (cyan sticks) shown with black dashes. (C) Overlay of cocrystal structures of 2-pyridine analogue (orange, PDB 9PV6 ^29^) and **41** (cyan PDB 9Z0D) bound to PL^Pro^ cartoon with key binding residues shown as lines and ligands shown as sticks. (D) 2mFo-DFc electron density maps of **41** (gray mesh, σ = 1.5) with thioether bond to Cys-111 shown as sticks. (E) 2D structures of crystallized PL^Pro^ ligands and their IC_50_ values.

## Chemistry

The synthesis of the final compounds involves three main parts: the core synthesis, middle linker synthesis, and warhead synthesis. A convergent synthetic route was devised whereby the core, linker and warhead portions were sequentially coupled to generate the required matrix libraries of final compounds. We previously reported the synthesis of spiro-chromone **I-5** and spiro-chromane **I-7** cores, which have been slightly modified to achieve gram-scale of key irreversible intermediates (Scheme 1).^29^

**Scheme 1:**
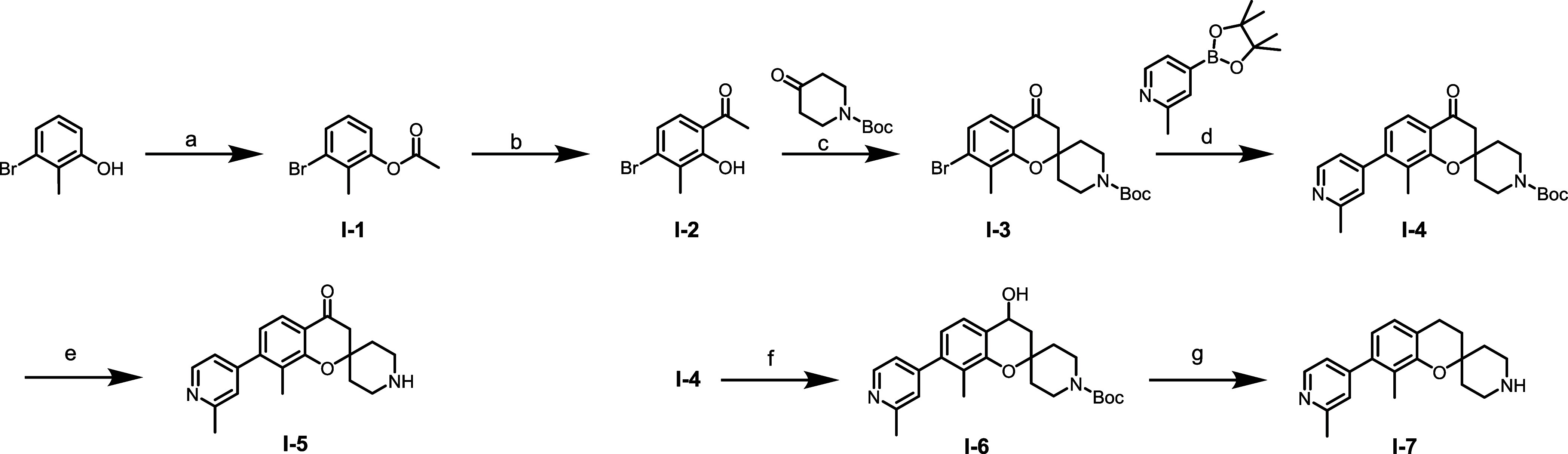
Synthesis of Spiro-chromone and Spiro-chromane Cores^a^

a Reagents and conditions. (a) Acetyl chloride, Et_3_N, DCM, rt; (b) AlCl_3_, sealed tube, 135 °C; (c) pyrrolidine, MeOH, 80 °C; (d) Pd­(dppf)­Cl_2_·DCM, Cs_2_CO_3_, dioxane, water, 90 °C; (e) TFA, DCM, 0 °C; (f) NaBH_4_, MeOH, 0 °C; (g) Et_3_SiH, TFA, 80 °C.

As described in Scheme 2, intermediate **I-8** was synthesized with good yield from phthalaldehyde via a Wittig reaction (Scheme 2A). Linkers **I-10** and **I-15** to **I-18** were generated by a Heck reaction of activated esters with 6-bromo substituted phenyl and pyridine-methyl alcohols followed by nucleophilic substitution with thionyl chloride in DMF (Scheme 2B,C). A Pd/C hydrogenation of **I-11** and subsequent chlorination using thionyl chloride furnished compound **I-20** in good yield (Scheme 2D). Alkylation of 1*H*-pyrrole-2-carbaldehyde and 1*H*-imidazole-2-carbaldehyde, with methyl 3-bromopropanoate afforded intermediates **I-21** and **I-22**, respectively (Scheme 2E). Reduction of the double bond in the middle linkers was carried out using H_2_ and 10% Pd/C.

**Scheme 2:**
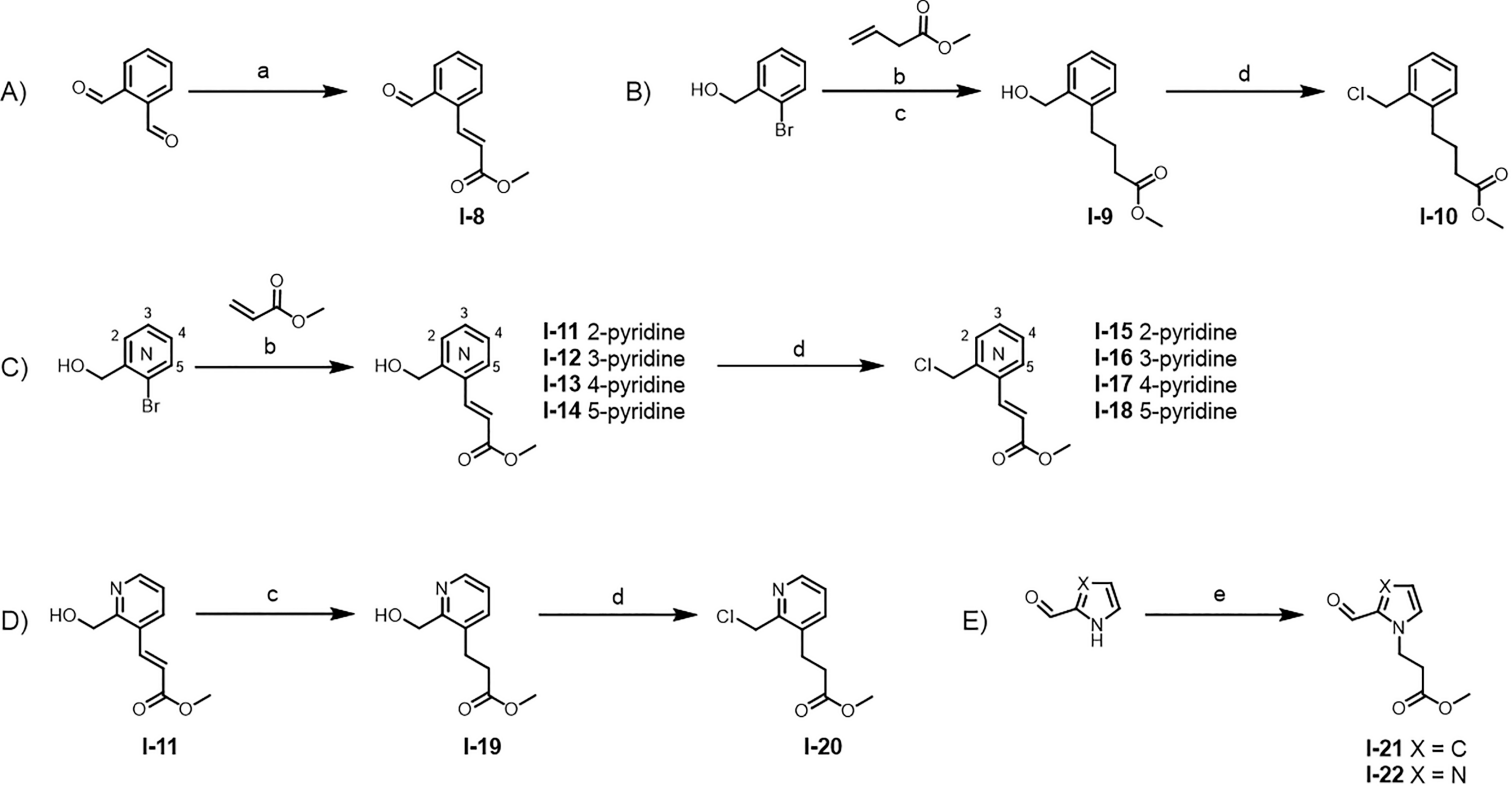
Synthesis of Key Linker Intermediates^a^

a Reagents and conditions. (a) Ethyl (triphenylphosphoranylidene)­acetate, DCM, rt; (b) Pd_2_(dba)_3_, tri-o-tolylphosphine, DIPEA, DMF, 90 °C; (c) H_2_, 10% Pd–C, EtOAc, rt; (d) SOCl_2_, DMF, 0 °C; (e) Methyl 3-bromopropanoate, NaH, DMF, 0 °C.

Key warhead intermediate **I-24** was obtained via an amide coupling of fumaric acid monomethyl ester with boc-hydrazine followed by a TFA deprotection (Scheme 3).

**Scheme 3:**
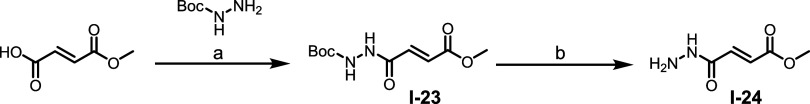
Synthesis of Warhead^a^

a Reagents and conditions. (a) HOBt, EDCI, DIPEA, DMF, 0 °C; (b) TFA, DCM, 0 °C.

The irreversible compounds **8**-**12**, having varied homologous alkyl chain lengths (n = 2 to 6) were synthesized by a sequential alkylation of key intermediate **I-5**, followed by hydrolysis and amide coupling. Synthesis of irreversible compounds **13** and **15**-**19** was achieved by a nucleophilic substitution of intermediate **I-5** with appropriate linker to afford **I-30** and **I-32** to **I-36**, followed by hydrolysis and EDCI/HOBt amide coupling. A reductive amination of middle linkers **I-8**, **I-21** and **I-22** with key intermediate **I-5** followed by hydrolysis and amide coupling furnished irreversible inhibitors **14**, **20**-**21** respectively (Scheme 4). Final compounds **22–27** were synthesized in similar fashion with Scheme 4 using their respective warheads.

**Scheme 4:**
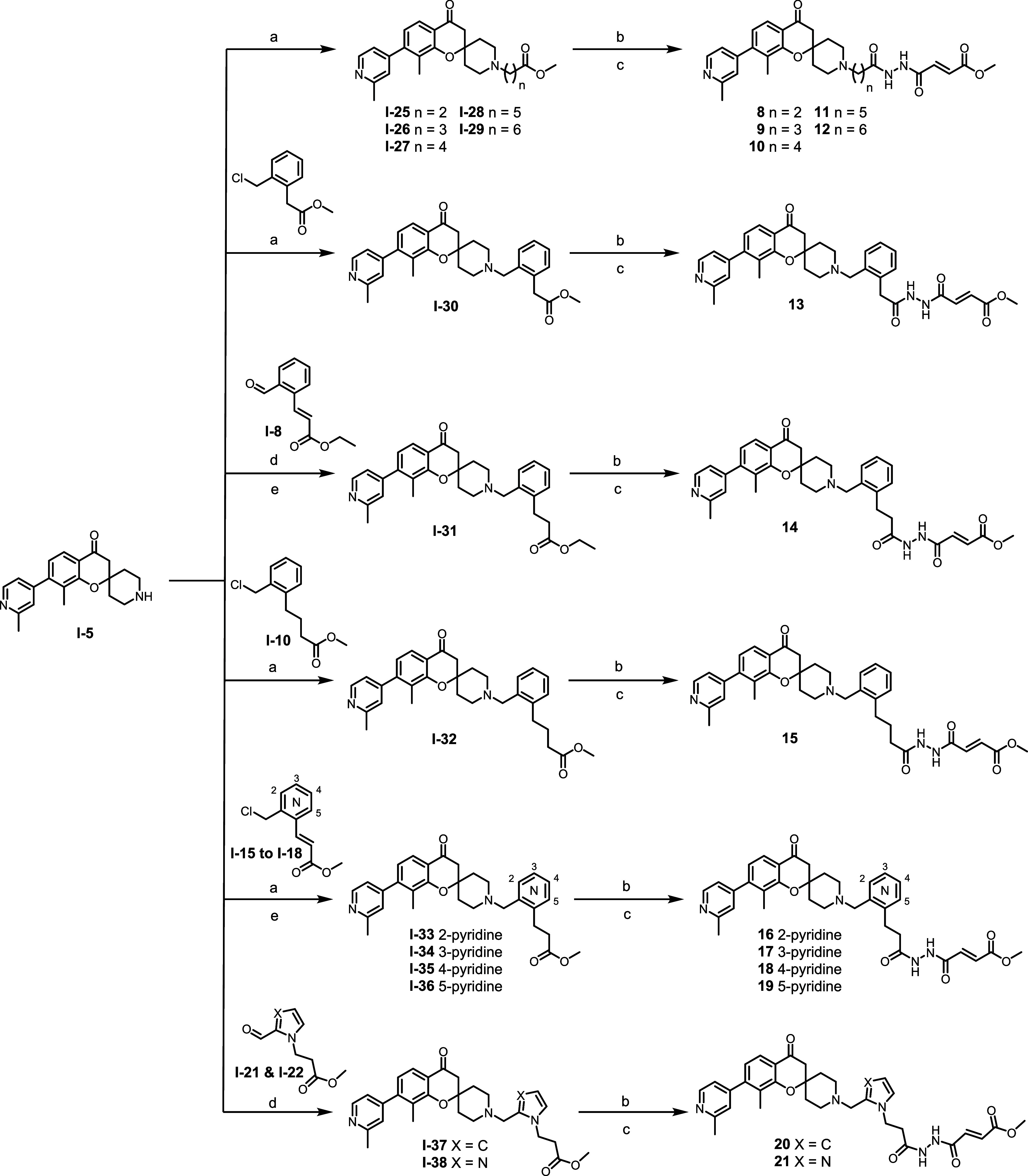
Synthesis of Irreversible Compounds **8**–**21** ^a^

a Reagents and conditions. (a) DIPEA, bromo alkyl acetate, KI, DCM, 35 °C; (b) 2 M NaOH aq., MeOH or THF, 30 °C; (c) **I-24**, HOBt, EDCI, DIPEA, DMF, rt; (d) STAB, AcOH, DCM, 50 °C; (e) H_2_, 10% Pd/C, EtOAc, rt.

Spiro-chromone inhibitors **28**-**38** were synthesized in good yields by hydrolysis of **16** followed by amide coupling with various substituted amines. Similar synthetic strategy was employed to generate the spiro-chromane irreversible inhibitors **39**-**50**. A sequential nucleophilic substitution of key intermediate **I-7** with linker **I-20**, base hydrolysis, amide coupling with warhead **I-24** and ester deprotection provided key intermediate **I-41**. A final amide coupling with various substituted amines produced compounds **39**-**50** (Scheme 5).

**Scheme 5:**
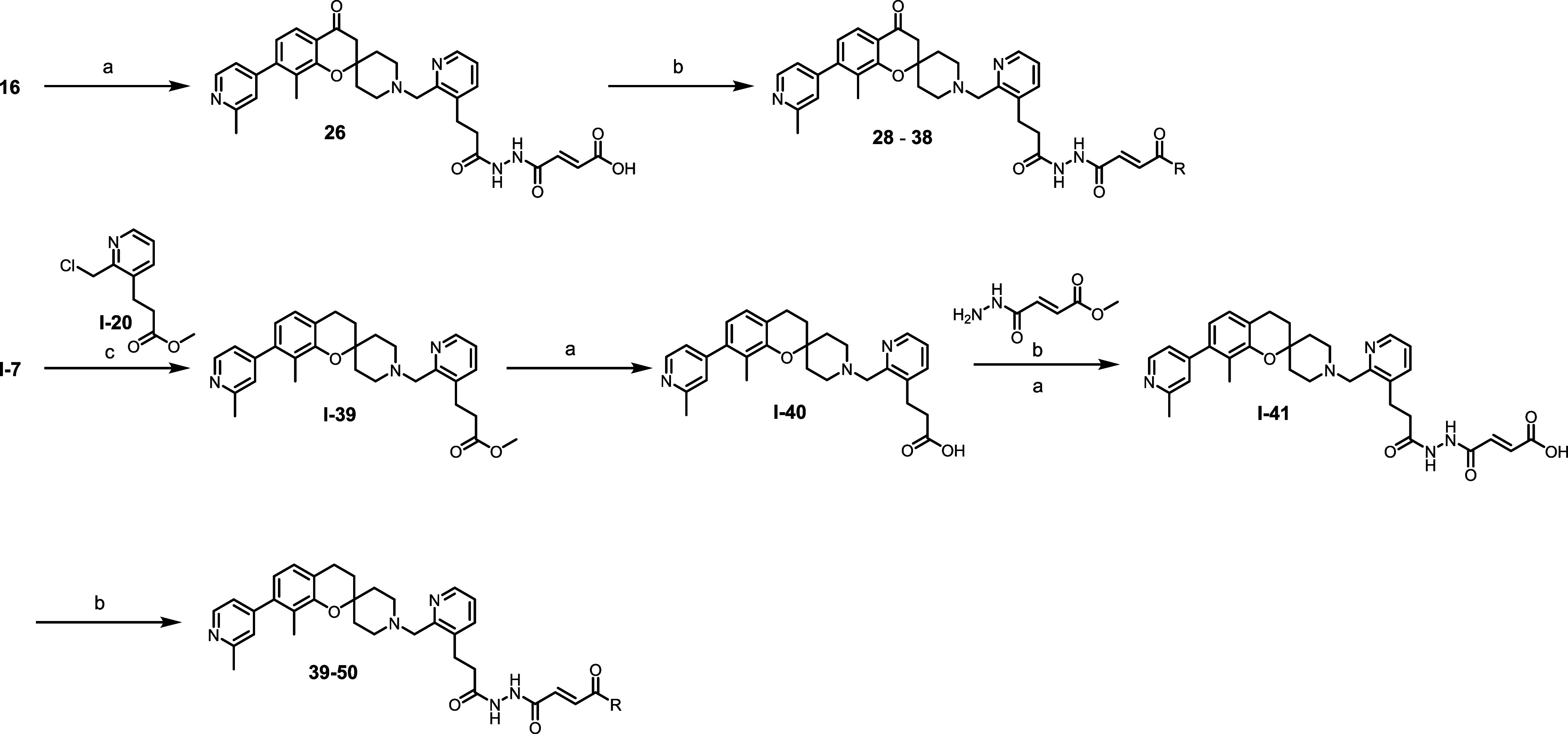
Synthesis of Carbonyl and Des-carbonyl Irreversible Acrylamides **28**–**50** ^a^

a Reagents and conditions. (a) 2 M NaOH aq., MeOH or THF, 30 °C; (b) substituted amines, HOBt, EDCI, DIPEA, DMF, rt; (c) DIPEA, KI, DCM, 35 °C.

## Conclusions

Despite its promise as an anticoronaviral target, there are currently no inhibitors of SARS-CoV-2 PL^Pro^ on the market or in clinical trials. Here we have discovered a novel class of irreversible spiro chromane PL^Pro^ inhibitors. Molecular dynamics revealed the key role of an intramolecular hydrogen bond between the 2-pyridyl linker and the piperidine nitrogen of the fragment core reducing conformational flexibility of the linker in solution and orienting the linker for expansion into the glycine channel. We obtained the first high resolution crystal structure of PL^Pro^ covalently labeled by a small molecule that identified a network of hydrogen bonds in the glycine channel and explained why the inclusion of the *N*,*N*’-diacetylhydrazine peptidomimetic is essential for maintaining high *in vitro* potency in enzymatic assays. However, this glycine channel binding moiety added two H-bond donors which probably cause a decrease in cell permeability that can hinder the *in vitro* potency in cellular antiviral activities. A challenge still remains for enhancing the permeability while maintaining all of the binding motifs. Previously disclosed methyl fumarates exhibited poor chemical and metabolic stability negatively impacting cellular activity. The conversion to the electron withdrawing acrylamides has enabled us to greatly improve the metabolic stability and increase the inhibitory activity. Optimization of the chromanone core via removal of the carbonyl further improved potency in biochemical and cellular assays with lead compound **45** having a IC_50_ of 0.059 at T = 60 min and an EC_50_ of 2.1 μM at 48 h post infection. Lead compound **45** exemplifies the first low nanomolar covalent inhibitor of PL^Pro^ not derived from GRL-0617 and represents a promising avenue for the discovery of a new class of therapeutic agents for the treatment of SARS-CoV-2 and potential future coronavirus outbreaks.

## Experimental Section

### General Information

The chemicals were purchased from commercial suppliers and used without prior purification unless otherwise stated. Proton nuclear magnetic resonance (^1^H NMR) spectra were recorded on a Bruker 400 MHz spectrometer. Coupling constants (*J*) were reported in hertz (Hz) and chemical shifts δ in ppm using the residual solvent line as a reference as 7.26 for Chlorofom (CDCl_3_), 2.50 for DMSO (DMSO-d_6_) and 2.92/2.75 for DMF (DMF-d_7_). Splitting patterns were designated using the following abbreviations: s, singlet; d, doublet; t, triplet; q, quartet; m, multiplet; br, broad; dd, doublet of doublet; dt, doublet of triplets. Thin layer chromatography (TLC) analyses were performed on Kieselgel 60 F254 glass plates precoated with a 0.25 mm thickness of silica gel. Liquid chromatography mass spectrometry (LC/MS) experiments were performed with the following parameters: Phenomenex Kinetex 2.6 μm XB-C18 100 Å, LC column (50 mm × 2.1 mm); method 2, 2 min, an autosampler, and a diode array detector, using a linear gradient of the binary solvent system of buffer A (Milli-Q H_2_O/MeCN/TFA, 95:5:0.1 v/v%) to buffer B (Milli-Q H_2_O/MeCN/TFA, 5:95:0.1 v/v%) with a flow rate of 1 mL/min. Silica gel chromatography was performed on Teledyne Isco CombiFlash systems using Redisep columns and EtOAc/Hexanes or MeOH/CH_2_Cl_2_ gradients. Preparative reversed-phase HPLC was performed on a Gilson instrument equipped with a Phenomenex Kinetex C18 column using a linear gradient of buffer A (Milli-Q H_2_O/MeCN/TFA, 95:5:0.1 v/v%) to buffer B (Milli-Q H_2_O/MeCN/TFA, 5:95:0.1 v/v%). All final compounds show ≥ 95% purity according to analytical LC/MS.

#### General Procedure A: EDC-Based Amide Coupling

EDC·HCl (1.5 equiv) was added portion-wise to a stirred suspension of fumarate acid (1.0 equiv), *tert*-butyl hydrazinecarboxylate (1.0 equiv) and DIPEA (3.0 equiv) in DCM at 0 °C for 30 min. The mixture was allowed to reach room temperature and then stirred for 4 h. Water was added and the mixture extracted with DCM. The combined organic layers were washed with brine, dried over Na_2_SO_4_, and concentrated under reduced pressure. Purification was conducted by flash column chromatography using a gradient of 0–10% MeOH in DCM.

#### General Procedure B: Boc Deprotection

A solution of the Boc protected amine (1.0 equiv) in DCM was cooled to 0 °C and added TFA (10.0 equiv) dropwise. The mixture was slowly allowed to reach room temperature. Upon reaction completion, solvent and excess TFA was removed under reduced pressure. The crude product was neutralized with saturated aqueous NaHCO_3_ at 0 °C and extracted with EtOAc. The combined organic layers were dried over Na_2_SO_4_, filtered and concentrated to dryness in vacuo. Purification was conducted by flash column chromatography using a gradient of 0–10% MeOH in DCM.

#### General Procedure C: NaH Mediated *N*-Alkylation with Alkyl Halides

NaH (2.5 equiv) was added to a solution of amine (1.0 equiv) in DMF at 0 °C. After the reaction mixture was stirred for 10 min, a solution of methyl 3-bromopropanoate (1.0 equiv) in DMF was added. Then the mixture was stirred at room temperature for 2 h. Upon reaction completion, saturated aqueous NaHCO_3_ was added and the mixture extracted with DCM. The combined organic layers were washed with brine, dried over Na_2_SO_4_, and concentrated under reduced pressure. The residue was purified by flash chromatography (Combi-flash Rf, MeOH/DCM = 0–10% gradient) to afford the desired product.

#### General Procedure D: Arylation and Alkylation of Alkenes/Heck Reaction

A mixture of aryl halide (1.0 equiv), methyl acrylate (1.5 equiv), Pd_2_(dba)_3_ (0.05 equiv), tri-o-tolylphosphane (0.10 equiv) and DIPEA (1.5 equiv) in DMF was stirred under argon at 100 °C for 16 h. The reaction mixture was cooled to room temperature, quenched with water and extracted with DCM. The combined organic layers were washed with brine, dried over Na_2_SO_4_, and concentrated. The residue was purified by flash chromatography (Combi-flash Rf, MeOH/DCM = 0–10% gradient) to afford the desired product.

#### General Procedure E: Formation of Alkyl Halides from Alcohols

To a stirred solution of alcohol (1.0 equiv) in DCM at 0 °C was added thionyl chloride (1.5 equiv). The reaction was then stirred for 1 h and then concentrated in vacuo. The crude product was neutralized with saturated aqueous NaHCO_3_ at 0 °C and extracted with EtOAc. The combined organic layers were dried over Na_2_SO_4_, filtered and concentrated. The residue was purified by flash chromatography (Combi-flash Rf, MeOH/DCM = 0–10% gradient) to afford the desired product.

#### General Procedure F: DIPEA and KI Mediated Amine N-Alkylation with Alkyl Halides

To a solution of amine (1.0 equiv), DIPEA (3.0 equiv) and KI (0.10 equiv) in DCM was added alkyl halide (1.2 equiv). The reaction was stirred at 40 °C overnight. Upon reaction completion, the mixture was concentrated and redissolved in EtOAc, washed with saturated aqueous NaHCO_3_, dried over Na_2_SO_4_, filtered and concentrated. The residue was purified by flash chromatography (Combi-flash Rf, MeOH/DCM = 0–10% gradient) to afford the desired product.

#### General Procedure G Base-Catalyzed Ester Hydrolysis

A solution of the ester (1.0 equiv) in MeOH was added aqueous 2 M NaOH (10.0 equiv). The mixture was stirred at 35 °C for 4 h. Upon reaction completion, the mixture was added water, pH adjusted appropriately with 1 M HCl, extracted with DCM and MeOH. The combined organic layers were dried over Na_2_SO_4_, filtered and concentrated. Intermediate compounds were used without further purification. Final compounds were purified by preparative reversed-phase HPLC (Phenomenex Gemini C18, MeCN/Milli-Q H_2_O = 10–65%, 0.1% TFA) followed by neutralization with saturated aqueous NaHCO_3_.

#### General Procedure H: EDC/HOBt-Based Amide Coupling

A solution of the carboxylic acid (1.0 equiv) in DMF was cooled to 0 °C and then added EDC·HCl (1.5 equiv), HOBt (1.5 equiv) and DIPEA (2.5 equiv). The mixture was stirred at 0 °C for 0.5 h then the appropriate amine (1.5 equiv) was added. The mixture was allowed to reach room temperature and stirred for 1 h. Upon reaction completion, the mixture was added water and extracted with EtOAc. The combined organic layers were dried over Na_2_SO_4_, filtered and concentrated. Intermediate compounds were purified by flash chromatography (Combi-flash Rf, MeOH/DCM = 0–10% gradient). Final compounds were purified by preparative reversed-phase HPLC (Phenomenex Gemini C18, MeCN/Milli-Q H_2_O = 10–65%, 0.1% TFA) followed by neutralization with saturated aqueous NaHCO_3_.

#### General Procedure I: Reduction of Alkenes

The alkene was dissolved in EtOAc containing 10% palladium on carbon and stirred under hydrogen (balloon pressure) for overnight. The mixture was filtered through Celite and the residue washed with EtOAc. The combined filtrates were concentrated to give the product. The compound was used without further purification.

#### General Procedure J: Alkylation of Piperidine Moiety by Reductive Amination

A solution of 8-methyl-7-(2-methylpyridin-4-yl)­spiro­[chromane-2,4’-piperidin]-4-one (1.0 equiv), aryl aldehyde (1.2 equiv) in DCM was added STAB (3.0 equiv) and 3 drops of acetic acid. The mixture was stirred at 55 °C for 2–16 h. At completion, the reaction was quenched with water, basified with aqueous 2 M NaOH and extracted with DCM and EtOAc. The combined organic layers were dried over Na_2_SO_4_, filtered and concentrated. The residue was purified by flash chromatography (Combi-flash Rf, MeOH/DCM = 0–10% gradient) to afford the desired product.

#### 3-Bromo-2-methylphenyl Acetate (I-1)

To a mixture of 3-bromo-2-methylphenol (12.5 g, 66.8 mmol, 1.0 equiv) and Et_3_N (18.6 mL, 134.0 mmol, 2.0 equiv) in DCM (200 mL) at 0 °C was added acetyl chloride (7.13 mL, 100.0 mmol, 1.5 equiv) dropwise. The reaction mixture was stirred at room temperature for 4 h and then quenched with ice water, diluted with DCM, washed with brine. The combined organic layers were dried over Na_2_SO_4_, filtered and concentrated under reduced pressure. The crude residue was purified by flash chromatography (Combi-flash Rf, EtOAc/hexanes = 0–10% gradient) to give the title compound (14.1 g, 90%). LCMS (ESI): *R*_t_ = 1.79 min, *m*/*z* = 229.0 [M + H]^+^.

#### 1-(4-Bromo-2-hydroxy-3-methylphenyl)­ethan-1-one (I-2)

Intermediate **I-1** (14.0 g, 61.1 mmol, 1.0 equiv) and AlCl_3_ (12.2 g, 91.7 mmol, 1.5 equiv) were mixed in a 250 mL round-bottom sealed tube. The resulting suspension was heated 135 °C for 4 h. the reaction mass was cooled to room temperature, diluted with EtOAc (50 mL), washed with water (60 mL) and brine, and the combined organic layers were dried over Na_2_SO_4_, filtered and concentrated under reduced pressure. The crude residue was purified by flash chromatography (Combi-flash Rf, EtOAc/hexanes = 0–20% gradient) to afford desired product (11.1 g, 79%). LCMS (ESI): *R*_t_ = 1.93 min, *m*/*z* = 229.0 [M + H]^+^.

#### *tert*-Butyl 7-Bromo-8-methyl-4-oxospiro­[chromane-2,4’-piperidine]-1’-carboxylate (I-3)

A mixture of **I-2** (11.0 g, 48.0 mmol, 1.0 equiv), *tert*-butyl 4-oxopiperidine-1-carboxylate (12.4 g, 62.4 mmol, 1.3 equiv) and pyrrolidine (6.01 mL, 72.0 mmol, 1.5 equiv) in MeOH (100 mL) was heated 80 °C for 4 h. The reaction mass was cooled to room temperature, diluted with DCM (100 mL), washed with water (50 mL) and brine, and the combined organic layers were dried over Na_2_SO_4_, filtered and concentrated under vacuum. The crude residue was purified by flash chromatography (Combi-flash Rf, EtOAc/hexanes = 0–10% gradient) to give desired product (19.0 g, 95%). LCMS (ESI): *R*_t_ = 2.23 min, *m*/*z* = 354.1 [M-56]^+^.

#### *tert*-Butyl 8-Methyl-7-(2-methylpyridin-4-yl)-4-oxospiro­[chromane-2,4’-piperidine]-1’-carboxylate (I-4)

To a stirred solution of **I-3** (15.0 g, 36.6 mmol, 1,0 equiv) and 2-methyl-4-(4,4,5,5-tetramethyl-1,3,2-dioxaborolan-2-yl)­pyridine (8.81 g, 40.2 mmol, 1.1.0 equiv) in dioxane (100 mL) and water (25 mL) was added Cs_2_CO_3_ (23.8 g, 73.1 mmol, 2.0 equiv) and Pd­(dppf)­Cl_2_·DCM (1.49 g, 1.83 mmol, 0.05 equiv). The mixture was sparged with argon for 10 min and then heated to 90 °C for 4 h. The reaction mixture was cooled to room temperature and diluted with EtOAc, washed with brine. The combined organic layers were dried over Na_2_SO_4_, filtered and concentrated under vacuum. The crude residue was purified by flash chromatography (Combi-flash Rf, EtOAc/hexanes = 0–10% gradient) to give desired product (12.0 g, 76%). LCMS (ESI): *R*_t_ = 1.60 min, *m*/*z* = 423.3 [M + H]^+^.

#### 8-Methyl-7-(2-methylpyridin-4-yl)­spiro­[chromane-2,4’-piperidin]-4-one (I-5)

General Procedure **B** was followed using **I-4** (10.0 g, 23.7 mmol, 1.0 equiv) and TFA (18.1 mL, 237.0 mmol, 10.0 equiv) to obtain the title compound (7.8 g, quantitative). LCMS (ESI): *R*_t_ = 1.06 min, *m*/*z* = 323.2 [M + H]^+^.

#### *tert*-Butyl 4-Hydroxy-8-methyl-7-(2-methylpyridin-4-yl)­spiro­[chromane-2,4’-piperidine]-1’-carboxylate (I-6)

To a stirred solution of **I-4** (5.0 g, 12.0 mmol, 1.0 equiv) in MeOH (50 mL) was added NaBH_4_ (1.1 g, 30.0 mmol, 2.0 equiv) portion-wise in ice cold condition, and the reaction mixture was allowed to warm up to room temperature and stirred for 1 h. The reaction mixture was added NH_4_Cl aqueous (20 mL) and extracted with EtOAc. The combined organic layers were concentrated in reduced pressure to afford desired product (5.0 g, quantitative), which was used in the next step without further purification. LCMS (ESI): *R*_t_ = 1.50 min, *m*/*z* = 425.3 [M + H]^+^.

#### 8-Methyl-7-(2-methylpyridin-4-yl)­spiro­[chromane-2,4’-piperidine] (I-7)

To a stirred solution of **I-6** (5.0 g, 12.0 mmol, 1.0 equiv) in TFA (30 mL) was added triethyl silane (2.8 mL, 18.0 mmol, 1.5 equiv). The mixture was stirred for 5 h at 80 °C. The reaction mass was cooled to room temperature and evaporated to dryness to 8-methyl-7-(2-methylpyridin-4-yl)­spiro­[chromane-2,4’-piperidine] as TFA salt. The crude product was neutralized with saturated aqueous NaHCO_3_ at 0 °C and extracted with DCM/MeOH. The combined organic layers were dried over Na_2_SO_4_, filtered and concentrated in reduced pressure to give the desired product (3.5 g, 95%). LCMS (ESI): *R*_t_ = 1.08 min, *m*/*z* = 309.3 [M + H]^+^.

#### Methyl (*E*)-3-(2-Formylphenyl)­acrylate (I-8)

A mixture of phthalaldehyde (3.0 g, 22.0 mmol, 1.0 equiv) and Ethyl (triphenylphosphoranylidene)­acetate (7.8 g, 22 mmol, 1.0 equiv) were dissolved in DCM and stirred at room temp for 0.5 h. The reaction mixture was diluted with EtOAc and washed with brine. The combined organic layers were dried over Na_2_SO_4_, filtered and concentrated under vacuum. The crude residue was purified by flash chromatography (Combi-flash Rf, EtOAc/hexanes = 0–20% gradient) to give desired product (2.99 g, 65%). LCMS (ESI): *R*_t_ = 1.57 min, *m*/*z* = 205.1 [M + H]^+^.

#### Methyl 4-(2-(Hydroxymethyl)­phenyl)­butanoate (I-9)

General Procedures D and I were followed sequentially using (2-bromophenyl)­methanol (50 mg, 0.1 mmol, 1.0 equiv) and methyl but-3-enoate (50 mg, 0.1 mmol, 1.0 equiv) to obtain the title compound (0.95 g, 84%). LCMS (ESI): *R*_t_ = 1.48 min, *m*/*z* = 191.2 [M-17]^+^.

#### Methyl 4-(2-(Chloromethyl)­phenyl)­butanoate (I-10)

General Procedure E was followed using **I-9** (0.90 g, 4.3 mmol, 1.0 equiv) and SOCl_2_ (0.63 mL, 8.6 mmol, 2.0 equiv) to obtain the title compound (34 mg, 95%). LCMS (ESI): *R*_t_ = 1.87 min, *m*/*z* = 189.2 [M-35]^+^.

#### Methyl (*E*)-3-(2-(Hydroxymethyl)­pyridin-3-yl)­acrylate (I-11)

General Procedure D was followed using (3-bromopyridin-2-yl)­methanol (20.0 g, 106.4 mmol, 1.0 equiv) and methyl but-3-enoate (13.7 g, 159.6 mmol, 1.5 equiv) to obtain the title compound (16.0 g, 77%). LCMS (ESI): *R*_t_ = 0.29 min, *m*/*z* = 194.1 [M + H]^+^.

#### Methyl (*E*)-3-(3-(Hydroxymethyl)­pyridin-4-yl)­acrylate (I-12)

General Procedure D was followed using (4-bromopyridin-3-yl)­methanol (0.50 g, 2.7 mmol, 1.0 equiv) and methyl but-3-enoate (0.34 g, 4.0 mmol, 1.5 equiv) to obtain the title compound (0.35 g, 69%). LCMS (ESI): R_t_ = 0.49 min, *m*/*z* = 194.1 [M + H]^+^.

#### Methyl (*E*)-3-(4-(Hydroxymethyl)­pyridin-3-yl)­acrylate (I-13)

General Procedure D was followed using (3-bromopyridin-4-yl)­methanol (0.50 g, 2.7 mmol, 1.0 equiv) and methyl but-3-enoate (0.34 g, 4.0 mmol, 1.5 equiv) to obtain the title compound (0.38 g, 73%). LCMS (ESI): R_t_ = 0.46 min, *m*/*z* = 194.1 [M + H]^+^.

#### Methyl (*E*)-3-(3-(Hydroxymethyl)­pyridin-2-yl)­acrylate (I-14)

General Procedure D was followed using (2-bromopyridin-3-yl)­methanol (0.50 g, 2.7 mmol, 1.0 equiv) and methyl but-3-enoate (0.34 g, 4.0 mmol, 1.5 equiv) to obtain the title compound (0.25 g, 49%). LCMS (ESI): R_t_ = 0.39 min, *m*/*z* = 194.1 [M + H]^+^.

#### Methyl (*E*)-3-(2-(Chloromethyl)­pyridin-3-yl)­acrylate (I-15)

General Procedure E was followed using **I-11** (2.00 g, 10.4 mmol, 1.0 equiv) and SOCl_2_ (1.5 mL, 20.7 mmol, 2.0 equiv) to obtain the title compound (1.9 g, 88%). LCMS (ESI): *R*_t_ = 1.24 min, *m*/*z* = 212.1 [M + H]^+^.

#### Methyl (*E*)-3-(3-(Chloromethyl)­pyridin-4-yl)­acrylate (I-16)

General Procedure E was followed using **I-12** (0.20 g, 1.0 mmol, 1.0 equiv) and SOCl_2_ (0.15 mL, 2.1 mmol, 2.0 equiv) to obtain the title compound (0.21 g, 95%). LCMS (ESI): *R*_t_ = 1.07 min, *m*/*z* = 212.1 [M + H]^+^.

#### Methyl (*E*)-3-(4-(Chloromethyl)­pyridin-3-yl)­acrylate (I-17)

General Procedure E was followed using **I-13** (0.20 g, 1.0 mmol, 1.0 equiv) and SOCl_2_ (0.15 mL, 2.1 mmol, 2.0 equiv) to obtain the title compound (0.22 g, 99%). LCMS (ESI): *R*_t_ = 1.10 min, *m*/*z* = 212.0 [M + H]^+^.

#### Methyl (*E*)-3-(3-(Chloromethyl)­pyridin-2-yl)­acrylate (I-18)

General Procedure E was followed using **I-14** (0.20 g, 1.0 mmol, 1.0 equiv) and SOCl_2_ (0.15 mL, 2.1 mmol, 2.0 equiv) to obtain the title compound (0.20 g, 91%). LCMS (ESI): *R*_t_ = 1.10 min, *m*/*z* = 212.1 [M + H]^+^.

#### Methyl 3-(2-(Hydroxymethyl)­pyridin-3-yl)­propanoate (I-19)

General Procedure I was followed using **I-11** (15.0 g, 77.6 mmol, 1.0 equiv) to obtain the title compound (15.2 g,100%). LCMS (ESI): *R*_t_ = 0.49 min, *m*/*z* = 196.2 [M + H]^+^.

#### Methyl 3-(2-(Chloromethyl)­pyridin-3-yl)­propanoate (I-20)

General Procedure E was followed using **I-19** (10.0 g, 51.2 mmol, 1.0 equiv) and SOCl_2_ (7.43 mL, 101.8 mmol, 2.0 equiv) to obtain the title compound (34 mg, 95%). LCMS (ESI): *R*_t_ = 0.89 min, *m*/*z* = 214.2 [M + H]^+^.

#### Methyl 3-(2-Formyl-1*H*-pyrrol-1-yl)­propanoate (I-21)

General Procedure C was followed using 1*H*-pyrrole-2-carbaldehyde (1.0 g, 10.5 mmol, 1.0 equiv) and methyl 3-bromopropanoate (2.11 g, 12.6 mmol, 1.2 equiv) to obtain the title compound (1.10 g, 56%). LCMS (ESI): *R*_t_ = 1.31 min, *m*/*z* = 182.1 [M + H]^+^.

#### Methyl 3-(2-Formyl-1*H*-imidazol-1-yl)­propanoate (I-22)

General Procedure C was followed using 1*H*-imidazole-2-carbaldehyde (1.0 g, 10.4 mmol, 1.0 equiv) and methyl 3-bromopropanoate (2.09 g, 12.5 mmol, 1.2 equiv) to obtain the title compound (0.82 g, 43%). LCMS (ESI): *R*_t_ = 0.27 min, *m*/*z* = 183.2 [M + H]^+^.

#### *tert*-Butyl (*E*)-2-(4-Methoxy-4-oxobut-2-enoyl)­hydrazine-1-carboxylate (I-23)

General Procedure A was followed using (*E*)-4-methoxy-4-oxobut-2-enoic acid (20.0 g, 153.7 mmol, 1.0 equiv) and *tert*-butyl hydrazinecarboxylate (26.4 g, 199.8 mmol, 1.3 equiv) to obtain the title compound (37.6 g, quantitative). LCMS (ESI): *R*_t_ = 1.24 min, *m*/*z* = 189.1 [M-56]^+^.

#### Methyl (*E*)-4-Hydrazineyl-4-oxobut-2-enoate (I-24)

General Procedure B was followed using **I-23** (20.0 g, 81.9 mmol, 1.0 equiv) to obtain the title compound (12.2 g, quantitative). LCMS (ESI): *R*_t_ = 0.28 min, *m*/*z* = 145.2 [M + H]^+^.

#### Methyl 3-(8-Methyl-7-(2-methylpyridin-4-yl)-4-oxospiro­[chromane-2,4’-piperidin]-1’-yl)­propanoate (I-25)

General Procedure F was followed using **I-5** (0.20 g, 0.62 mmol, 1.0 equiv) to obtain the title compound (0.22 g, 84%). LCMS (ESI): *R*_t_ = 1.09 min, *m*/*z* = 409.2 [M + H]^+^.

#### Methyl 4-(8-Methyl-7-(2-methylpyridin-4-yl)-4-oxospiro­[chromane-2,4’-piperidin]-1’-yl)­butanoate (I-26)

General Procedure F was followed using **I-5** (0.20 g, 0.62 mmol, 1.0 equiv) to obtain the title compound (0.24 g, 92%). LCMS (ESI): *R*_t_ = 1.07 min, *m*/*z* = 423.1 [M + H]^+^.

#### Methyl 5-(8-Methyl-7-(2-methylpyridin-4-yl)-4-oxospiro­[chromane-2,4’-piperidin]-1’-yl)­pentanoate (I-27)

General Procedure F was followed using **I-5** (0.20 g, 0.62 mmol, 1.0 equiv) to obtain the title compound (0.17 g, 64%). LCMS (ESI): *R*_t_ = 1.10 min, *m*/*z* = 437.1 [M + H]^+^.

#### Methyl 6-(8-Methyl-7-(2-methylpyridin-4-yl)-4-oxospiro­[chromane-2,4’-piperidin]-1’-yl)­hexanoate (I-28)

General Procedure F was followed using **I-5** (0.20 g, 0.62 mmol, 1.0 equiv) to obtain the title compound (0.15 g, 52%). LCMS (ESI): *R*_t_ = 1.13 min, *m*/*z* = 451.2 [M + H]^+^.

#### Methyl 7-(8-Methyl-7-(2-methylpyridin-4-yl)-4-oxospiro­[chromane-2,4’-piperidin]-1’-yl)­heptanoate (I-29)

General Procedure F was followed using **I-5** (0.20 g, 0.62 mmol, 1.0 equiv) to obtain the title compound (0.25 g, 88%). LCMS (ESI): *R*_t_ = 1.13 min, *m*/*z* = 465.1 [M + H]^+^.

#### Methyl (*E*)-4-(2-(3-(8-Methyl-7-(2-methylpyridin-4-yl)-4-oxospiro­[chromane-2,4’-piperidin]-1’-yl)­propanoyl)­hydrazineyl)-4-oxobut-2-enoate (8)

General Procedures G and H were followed sequentially using **I-25** (0.10 g, 0.25 mmol, 1.0 equiv) to obtain the title compound (28.0 mg, 21%). ^1^H NMR (400 MHz, DMSO) δ 10.72 (s, 1H), 10.44 (s, 1H), 8.52 (d, *J* = 5.1 Hz, 1H), 7.66 (d, *J* = 8.1 Hz, 1H), 7.26 (s, 1H), 7.19 (dd, *J* = 5.1, 1.7 Hz, 1H), 7.07 (d, *J* = 15.6 Hz, 1H), 6.91 (d, *J* = 8.1 Hz, 1H), 6.67 (d, *J* = 15.6 Hz, 1H), 3.74 (s, 3H), 2.83 (s, 2H), 2.70 (dd, *J* = 12.0, 9.2 Hz, 2H), 2.62 (t, *J* = 6.9 Hz, 2H), 2.53 (s, 3H), 2.41–2.30 (m, 4H), 2.16 (s, 3H), 1.95 (d, *J* = 13.6 Hz, 2H), 1.79–1.67 (m, 2H). LCMS (ESI): *R*_t_ = 1.10 min, *m*/*z* = 521.2 [M + H]^+^.

#### Methyl (*E*)-4-(2-(4-(8-Methyl-7-(2-methylpyridin-4-yl)-4-oxospiro­[chromane-2,4’-piperidin]-1’-yl)­butanoyl)­hydrazineyl)-4-oxobut-2-enoate (9)

General Procedures G and H were followed sequentially using **I-26** (0.10 g, 0.24 mmol, 1.0 equiv) to obtain the title compound (29.0 mg, 22%) ^1^H NMR (400 MHz, DMSO) δ 10.54 (s, 1H), 10.15 (s, 1H), 8.52 (d, *J* = 5.1 Hz, 1H), 7.65 (d, *J* = 8.1 Hz, 1H), 7.26 (s, 1H), 7.18 (dd, *J* = 5.2, 1.7 Hz, 1H), 7.06 (d, *J* = 15.6 Hz, 1H), 6.91 (d, *J* = 8.1 Hz, 1H), 6.66 (d, *J* = 15.6 Hz, 1H), 3.74 (s, 3H), 2.82 (s, 2H), 2.64 (d, *J* = 11.3 Hz, 2H), 2.53 (s, 3H), 2.36–2.23 (m, 4H), 2.20 (d, *J* = 7.3 Hz, 2H), 2.15 (d, *J* = 2.1 Hz, 3H), 1.97–1.85 (m, 2H), 1.78–1.60 (m, 4H). LCMS (ESI): *R*_t_ = 1.07 min, *m*/*z* = 535.0 [M + H]^+^.

#### Methyl (*E*)-4-(2-(5-(8-Methyl-7-(2-methylpyridin-4-yl)-4-oxospiro­[chromane-2,4’-piperidin]-1’-yl)­pentanoyl)­hydrazineyl)-4-oxobut-2-enoate (10)

General Procedures G and H were followed sequentially using **I-27** (0.10 g, 0.24 mmol, 1.0 equiv) to obtain the title compound (19.0 mg, 15%). ^1^H NMR (400 MHz, DMSO) δ 10.51 (s, 1H), 10.10 (s, 1H), 8.52 (d, *J* = 5.1 Hz, 1H), 7.65 (d, *J* = 8.0 Hz, 1H), 7.26 (s, 1H), 7.19 (dd, *J* = 5.2, 1.7 Hz, 1H), 7.05 (d, *J* = 15.6 Hz, 1H), 6.91 (d, *J* = 8.1 Hz, 1H), 6.67 (d, *J* = 15.6 Hz, 1H), 3.74 (s, 3H), 2.82 (s, 2H), 2.64 (d, *J* = 11.3 Hz, 2H), 2.53 (s, 3H), 2.35–2.20 (m, 4H), 2.16 (d, *J* = 10.0 Hz, 5H), 1.93 (d, *J* = 13.5 Hz, 2H), 1.72 (t, *J* = 13.4 Hz, 2H), 1.53 (q, *J* = 7.3 Hz, 2H), 1.46 (q, *J* = 7.3 Hz, 2H). LCMS (ESI): *R*_t_ = 1.00 min, *m*/*z* = 549.1 [M + H]^+^.

#### Methyl (*E*)-4-(2-(6-(8-Methyl-7-(2-methylpyridin-4-yl)-4-oxospiro­[chromane-2,4’-piperidin]-1’-yl)­hexanoyl)­hydrazineyl)-4-oxobut-2-enoate (11)

General Procedures G and H were followed sequentially using **I-28** (0.10 g, 0.23 mmol, 1.0 equiv) to obtain the title compound (24.0 mg, 19%). ^1^H NMR (400 MHz, DMSO) δ 10.50 (s, 1H), 10.09 (s, 1H), 8.52 (d, *J* = 5.1 Hz, 1H), 7.65 (d, *J* = 8.0 Hz, 1H), 7.26 (s, 1H), 7.19 (dd, *J* = 5.0, 1.7 Hz, 1H), 7.05 (d, *J* = 15.6 Hz, 1H), 6.91 (d, *J* = 8.1 Hz, 1H), 6.65 (d, *J* = 15.6 Hz, 1H), 3.74 (s, 3H), 2.82 (s, 2H), 2.64 (d, *J* = 11.7 Hz, 2H), 2.53 (s, 3H), 2.27 (dt, *J* = 14.9, 8.8 Hz, 4H), 2.14 (d, *J* = 5.5 Hz, 5H), 1.93 (d, *J* = 13.6 Hz, 2H), 1.72 (t, *J* = 12.6 Hz, 2H), 1.53 (p, *J* = 7.2 Hz, 2H), 1.43 (t, *J* = 7.5 Hz, 2H), 1.33–1.24 (m, 2H). LCMS (ESI): *R*_t_ = 1.02 min, *m*/*z* = 563.1 [M + H]^+^.

#### Methyl (*E*)-4-(2-(7-(8-Methyl-7-(2-methylpyridin-4-yl)-4-oxospiro­[chromane-2,4’-piperidin]-1’-yl)­heptanoyl)­hydrazineyl)-4-oxobut-2-enoate (12)

General Procedures G and H were followed sequentially using **I-29** (0.10 g, 0.22 mmol, 1.0 equiv) to obtain the title compound (15.0 mg, 12%). ^1^H NMR (400 MHz, DMSO) δ 10.51 (s, 1H), 10.10 (s, 1H), 8.52 (d, *J* = 5.1 Hz, 1H), 7.65 (d, *J* = 8.0 Hz, 1H), 7.26 (s, 1H), 7.20–7.16 (m, 1H), 7.05 (d, *J* = 15.6 Hz, 1H), 6.91 (d, *J* = 8.1 Hz, 1H), 6.67 (d, *J* = 15.6 Hz, 1H), 3.74 (s, 3H), 2.82 (s, 2H), 2.70–2.61 (m, 2H), 2.53 (s, 3H), 2.27 (dt, *J* = 14.0, 7.2 Hz, 4H), 2.15 (s, 5H), 1.93 (d, *J* = 13.7 Hz, 2H), 1.72 (t, *J* = 12.5 Hz, 2H), 1.47 (d, *J* = 39.4 Hz, 4H), 1.32–1.21 (m, 4H). LCMS (ESI): *R*_t_ = 1.14 min, *m*/*z* = 577.1 [M + H]^+^.

#### Methyl 2-(2-((8-Methyl-7-(2-methylpyridin-4-yl)-4-oxospiro­[chromane-2,4’-piperidin]-1’-yl)­methyl)­phenyl)­acetate (I-30)

General Procedure F was followed sequentially using **I-5** (0.20 g, 0.62 mmol, 1.0 equiv) and methyl 2-(2-(chloromethyl)­phenyl)­acetate (0.15 g, 0.74 mmol, 1.2 equiv) to obtain the title compound (0.29 g, 95%). LCMS (ESI): *R*_t_ = 1.20 min, *m*/*z* = 485.1 [M + H]^+^.

#### Methyl (*E*)-4-(2-(2-(2-((8-Methyl-7-(2-methylpyridin-4-yl)-4-oxospiro­[chromane-2,4’-piperidin]-1’-yl)­methyl)­phenyl)­acetyl)­hydrazineyl)-4-oxobut-2-enoate (13)

General Procedures G and H were followed sequentially using **I-30** (0.10 g, 0.21 mmol, 1.0 equiv) to obtain the title compound (57.0 mg, 45%). ^1^H NMR (400 MHz, DMSO) δ 10.81 (s, 1H), 8.53 (d, *J* = 5.1 Hz, 1H), 7.64 (d, *J* = 8.1 Hz, 1H), 7.31–7.13 (m, 6H), 7.06 (d, *J* = 15.5 Hz, 1H), 6.91 (d, *J* = 8.1 Hz, 1H), 6.67 (d, *J* = 15.5 Hz, 1H), 3.73 (s, 3H), 3.69 (s, 2H), 3.58 (s, 2H), 2.77 (s, 2H), 2.65 (d, *J* = 11.2 Hz, 2H), 2.54 (s, 3H), 2.40 (t, *J* = 11.1 Hz, 2H), 2.17 (s, 3H), 1.91 (d, *J* = 13.7 Hz, 2H), 1.75–1.64 (m, 2H). LCMS (ESI): *R*_t_ = 1.20 min, *m*/*z* = 597.1 [M + H]^+^.

#### Ethyl 3-(2-((8-Methyl-7-(2-methylpyridin-4-yl)-4-oxospiro­[chromane-2,4’-piperidin]-1’-yl)­methyl)­phenyl)­propanoate (I-31)

General Procedures J and I were followed sequentially using **I-5** (0.20 g, 0.62 mmol, 1.0 equiv) to obtain the title compound (0.28 g, 89%). LCMS (ESI): *R*_t_ = 1.29 min, *m*/*z* = 511.1 [M + H]^+^.

#### Methyl (*E*)-4-(2-(3-(2-((8-Methyl-7-(2-methylpyridin-4-yl)-4-oxospiro­[chromane-2,4’-piperidin]-1’-yl)­methyl)­phenyl)­propanoyl)­hydrazineyl)-4-oxobut-2-enoate (14)

General Procedure G and H were followed sequentially using **I-31** (0.10 g, 0.20 mmol, 1.0 equiv) to obtain the title compound (39.0 mg, 35%). ^1^H NMR (400 MHz, DMSO) δ 10.54 (s, 1H), 10.17 (s, 1H), 8.53 (d, *J* = 5.1 Hz, 1H), 7.65 (d, *J* = 8.1 Hz, 1H), 7.29–7.11 (m, 6H), 7.07 (d, *J* = 15.6 Hz, 1H), 6.91 (d, *J* = 8.0 Hz, 1H), 6.68 (d, *J* = 15.6 Hz, 1H), 3.75 (s, 3H), 3.50 (s, 2H), 2.98–2.88 (t, 2H), 2.84 (s, 2H), 2.63 (s, 2H), 2.54 (s, 3H), 2.45–2.51 (t, 2H), 2.37 (t, *J* = 11.2 Hz, 2H), 2.19 (s, 3H), 1.94 (d, *J* = 13.6 Hz, 2H), 1.73 (t, *J* = 12.7 Hz, 2H). LCMS (ESI): *R*_t_ = 1.20 min, *m*/*z* = 611.1 [M + H]^+^.

#### Methyl 4-(2-((8-Methyl-7-(2-methylpyridin-4-yl)-4-oxospiro­[chromane-2,4’-piperidin]-1’-yl)­methyl)­phenyl)­butanoate (I-32)

General Procedure F was followed using **I-5** (0.10 g, 0.31 mmol, 1.0 equiv) to obtain the title compound (0.10 g, 65%). LCMS (ESI): *R*_t_ = 1.39 min, *m*/*z* = 513.3 [M + H]^+^.

#### Methyl (*E*)-4-(2-(4-(2-((8-Methyl-7-(2-methylpyridin-4-yl)-4-oxospiro­[chromane-2,4’-piperidin]-1’-yl)­methyl)­phenyl)­butanoyl)­hydrazineyl)-4-oxobut-2-enoate (15)

General Procedures G and H were followed sequentially using **I-32** (0.10 g, 0.20 mmol, 1.0 equiv) to obtain the title compound (38.0 mg, 31%). ^1^H NMR (400 MHz, DMSO) δ 10.51 (s, 1H), 10.09 (s, 1H), 8.53 (d, *J* = 5.0 Hz, 1H), 7.64 (d, *J* = 8.0 Hz, 1H), 7.31–7.09 (m, 6H), 7.06 (d, *J* = 15.5 Hz, 1H), 6.94–6.88 (m, 1H), 6.57 (d, *J* = 15.5 Hz, 1H), 3.72 (s, 3H), 3.48 (s, 2H), 2.82 (s, 2H), 2.71–2.60 (m, 4H), 2.54 (s, 3H), 2.36 (t, *J* = 11.2 Hz, 2H), 2.24 (d, *J* = 7.4 Hz, 2H), 2.19 (s, 3H), 1.92 (d, *J* = 13.3 Hz, 2H), 1.86–1.76 (m, 2H), 1.76–1.64 (m, 2H). LCMS (ESI): *R*_t_ = 1.30 min, *m*/*z* = 625.3 [M + H]^+^.

#### Methyl 3-(2-((8-Methyl-7-(2-methylpyridin-4-yl)-4-oxospiro­[chromane-2,4’-piperidin]-1’-yl)­methyl)­pyridin-3-yl)­propanoate (I-33)

General Procedures F and I were followed sequentially using **I-5** (5.0 g, 15.5 mmol, 1.0 equiv) to obtain the title compound (5.8 g, 75%). LCMS (ESI): *R*_t_ = 1.14 min, *m*/*z* = 500.1 [M + H]^+^.

#### Methyl 3-(3-((8-Methyl-7-(2-methylpyridin-4-yl)-4-oxospiro­[chromane-2,4’-piperidin]-1’-yl)­methyl)­pyridin-4-yl)­propanoate (I-34)

General Procedures F and I were followed sequentially using **I-5** (0.20 g, 0.62 mmol, 1.0 equiv) to obtain the title compound (0.27 g, 88%). LCMS (ESI): *R*_t_ = 1.09 min, *m*/*z* = 500.1 [M + H]^+^.

#### Methyl 3-(4-((8-Methyl-7-(2-methylpyridin-4-yl)-4-oxospiro­[chromane-2,4’-piperidin]-1’-yl)­methyl)­pyridin-3-yl)­propanoate (I-35)

General Procedures F and I were followed sequentially using **I-5** (0.20 g, 0.62 mmol, 1.0 equiv) to obtain the title compound (0.26 g, 85%). LCMS (ESI): *R*_t_ = 1.09 min, *m*/*z* = 500.1 [M + H]^+^.

#### Methyl 3-(3-((8-Methyl-7-(2-methylpyridin-4-yl)-4-oxospiro­[chromane-2,4’-piperidin]-1’-yl)­methyl)­pyridin-2-yl)­propanoate (I-36)

General Procedures F and I were followed sequentially using **I-5** (0.20 g, 0.62 mmol, 1.0 equiv) to obtain the title compound (0.24 g, 79%). LCMS (ESI): *R*_t_ = 1.09 min, *m*/*z* = 500.1 [M + H]^+^.

#### Methyl (*E*)-4-(2-(3-(2-((8-Methyl-7-(2-methylpyridin-4-yl)-4-oxospiro­[chromane-2,4’-piperidin]-1’-yl)­methyl)­pyridin-3-yl)­propanoyl)­hydrazineyl)-4-oxobut-2-enoate (16)

General Procedure G and H were followed sequentially using **I-33** (0.10 g, 0.21 mmol, 1.0 equiv) to obtain the title compound (52.0 mg, 45%). ^1^H NMR (400 MHz, DMSO) δ 10.69 (s, 1H), 10.20 (s, 1H), 8.53 (d, *J* = 5.1 Hz, 1H), 8.05–7.99 (m, 1H), 7.68–7.62 (m, 1H), 7.27 (s, 1H), 7.20 (d, *J* = 5.3 Hz, 1H), 7.14 (d, *J* = 15.6 Hz, 1H), 7.07 (d, *J* = 15.6 Hz, 1H), 6.91 (d, *J* = 8.1 Hz, 1H), 6.68 (d, *J* = 15.8 Hz, 2H), 3.75 (d, *J* = 1.4 Hz, 5H), 2.99 (t, *J* = 7.8 Hz, 2H), 2.81 (s, 2H), 2.59 (s, 4H), 2.54 (s, 3H), 2.44 (d, *J* = 12.5 Hz, 2H), 2.18 (s, 3H), 1.92 (d, *J* = 13.4 Hz, 2H), 1.69 (q, *J* = 12.9 Hz, 2H). LCMS (ESI): *R*_t_ = 1.07 min, *m*/*z* = 612.1 [M + H]^+^.

#### Ethyl (*E*)-4-(2-(3-(3-((8-Methyl-7-(2-methylpyridin-4-yl)-4-oxospiro­[chromane-2,4’-piperidin]-1’-yl)­methyl)­pyridin-4-yl)­propanoyl)­hydrazineyl)-4-oxobut-2-enoate (17)

General Procedures G and H were followed sequentially using **I-34** (0.10 g, 0.20 mmol, 1.0 equiv) to obtain the title compound (34 mg, 29%). ^1^H NMR (400 MHz, DMSO) δ 10.64 (s, 1H), 10.30 (s, 1H), 8.59 (d, *J* = 5.1 Hz, 1H), 8.45–8.37 (m, 1H), 7.69 (d, *J* = 8.0 Hz, 1H), 7.60 (d, *J* = 7.6 Hz, 1H), 7.39–7.19 (m, 3H), 7.11 (dd, *J* = 15.6, 6.9 Hz, 1H), 6.97 (d, *J* = 8.1 Hz, 1H), 6.73 (dd, *J* = 15.6, 7.9 Hz, 1H), 3.78 (s, 3H), 3.61 (s, 2H), 2.99 (d, *J* = 12.9 Hz, 4H), 2.79–2.66 (m, 4H), 2.59 (s, 3H), 2.51–2.35 (m, 2H), 2.32–2.13 (m, 3H), 1.97 (s, 2H), 1.88 (s, 2H). LCMS (ESI): *R*_t_ = 1.21 min, *m*/*z* = 612.1 [M + H]^+^.

#### Methyl (*E*)-4-(2-(3-(4-((8-Methyl-7-(2-methylpyridin-4-yl)-4-oxospiro­[chromane-2,4’-piperidin]-1’-yl)­methyl)­pyridin-3-yl)­propanoyl)­hydrazineyl)-4-oxobut-2-enoate (18)

General Procedures G and H were followed sequentially using **I-35** (0.10 g, 0.20 mmol, 1.0 equiv) to obtain the title compound (18.0 mg, 16%). ^1^H NMR (400 MHz, DMSO) δ 10.21 (s, 1H), 9.95 (s, 1H), 8.59–8.49 (m, 1H), 8.46–8.30 (m, 2H), 7.65 (d, *J* = 8.0 Hz, 1H), 7.57 (d, *J* = 7.9 Hz, 1H), 7.30 (d, *J* = 5.0 Hz, 1H), 7.26 (d, *J* = 7.1 Hz, 1H), 7.19 (s, 1H), 7.03 (d, *J* = 15.5 Hz, 1H), 6.91 (d, *J* = 8.0 Hz, 1H), 3.69 (s, 3H), 3.55 (s, 2H), 2.98–2.78 (m, 4H), 2.69 (s, 2H), 2.62 (s, 2H), 2.55–2.52 (m, 3H), 2.42 (s, 2H), 2.19 (s, 3H), 2.10–2.03 (m, 2H), 1.95 (d, *J* = 12.2 Hz, 2H). LCMS (ESI): *R*_t_ = 1.12 min, *m*/*z* = 612.1 [M + H]^+^.

#### Methyl (*E*)-4-(2-(3-(3-((8-Methyl-7-(2-methylpyridin-4-yl)-4-oxospiro­[chromane-2,4’-piperidin]-1’-yl)­methyl)­pyridin-2-yl)­propanoyl)­hydrazineyl)-4-oxobut-2-enoate (19)

General Procedures G and H were followed sequentially using **I-36** (0.10 g, 0.20 mmol, 1.0 equiv) to obtain the title compound (12.0 mg, 15%). ^1^H NMR (400 MHz, DMSO) δ 10.60 (s, 1H), 10.23 (s, 1H), 8.53 (d, *J* = 5.1 Hz, 1H), 8.40–8.34 (m, 1H), 7.96 (t, *J* = 8.9 Hz, 1H), 7.65 (d, *J* = 8.2 Hz, 1H), 7.25 (s, 1H), 7.18 (s, 1H), 7.10–7.03 (m, 1H), 6.91 (d, *J* = 8.0 Hz, 1H), 6.88–6.80 (m, 1H), 6.67 (d, *J* = 15.2 Hz, 1H), 3.74 (d, *J* = 2.1 Hz, 5H), 3.07 (t, 2H), 3.00–2.86 (m, 4H), 2.71–2.64 (m, 2H), 2.53 (s, 3H), 2.36–2.26 (m, 2H), 2.19 (s, 2H), 2.08 (d, *J* = 4.2 Hz, 5H). LCMS (ESI): *R*_t_ = 1.13 min, *m*/*z* = 612.1 [M + H]^+^.

#### Methyl 3-(2-((8-Methyl-7-(2-methylpyridin-4-yl)-4-oxospiro­[chromane-2,4’-piperidin]-1’-yl)­methyl)-1*H*-pyrrol-1-yl)­propanoate (I-37)

General Procedure J was followed using **I-5** (0.30 g, 0.93 mmol, 1.0 equiv) to obtain the title compound (0.30 g, 66%). LCMS (ESI): *R*_t_ = 1.30 min, *m*/*z* = 488.1 [M + H]^+^.

#### Methyl 3-(2-((8-Methyl-7-(2-methylpyridin-4-yl)-4-oxospiro­[chromane-2,4’-piperidin]-1’-yl)­methyl)-1*H*-imidazol-1-yl)­propanoate (I-38)

General Procedure J was followed using **I-5** (0.30 g, 0.93 mmol, 1.0 equiv) to obtain the title compound (0.25 g, 55%). LCMS (ESI): *R*_t_ = 1.15 min, *m*/*z* = 489.1 [M + H]^+^.

#### Methyl (*E*)-4-(2-(3-(2-((8-Methyl-7-(2-methylpyridin-4-yl)-4-oxospiro­[chromane-2,4’-piperidin]-1’-yl)­methyl)-1*H*-pyrrol-1-yl)­propanoyl)­hydrazineyl)-4-oxobut-2-enoate (20)

General Procedures G and H were followed sequentially using **I-37** (0.10 g, 0.21 mmol, 1.0 equiv) to obtain the title compound (31.0 mg, 29%). ^1^H NMR (400 MHz, DMSO) δ 10.51 (s, 1H), 10.24 (s, 1H), 8.53 (d, *J* = 5.1 Hz, 1H), 7.65 (d, *J* = 8.0 Hz, 1H), 7.26 (s, 1H), 7.19 (dd, *J* = 5.1, 1.7 Hz, 1H), 7.07 (d, *J* = 15.6 Hz, 1H), 6.91 (d, *J* = 8.1 Hz, 1H), 6.75–6.63 (m, 2H), 5.91–5.79 (m, 2H), 4.16 (t, *J* = 7.1 Hz, 2H), 3.75 (s, 3H), 3.43 (s, 2H), 2.83 (s, 2H), 2.67 (dt, *J* = 15.6, 8.7 Hz, 4H), 2.53 (s, 3H), 2.28 (t, *J* = 11.5 Hz, 2H), 2.17 (s, 3H), 1.94 (d, *J* = 13.5 Hz, 2H), 1.72 (td, *J* = 13.3, 4.4 Hz, 2H). LCMS (ESI): *R*_t_ = 1.56 min, *m*/*z* = 600.1 [M + H]^+^.

#### Methyl (*E*)-4-(2-(3-(2-((8-Methyl-7-(2-methylpyridin-4-yl)-4-oxospiro­[chromane-2,4’-piperidin]-1’-yl)­methyl)-1*H*-imidazol-1-yl)­propanoyl)­hydrazineyl)-4-oxobut-2-enoate (21)

General Procedures G and H were followed sequentially using **I-38** (0.10 g, 0.21 mmol, 1.0 equiv) to obtain the title compound (29.0 mg, 33%). ^1^H NMR (400 MHz, DMSO) δ 10.61 (s, 1H), 10.28 (s, 1H), 8.53 (d, *J* = 5.1 Hz, 1H), 7.65 (d, *J* = 8.1 Hz, 1H), 7.27 (d, *J* = 1.6 Hz, 1H), 7.19 (dd, *J* = 5.1, 1.7 Hz, 1H), 7.13–7.03 (m, 2H), 6.91 (d, *J* = 8.1 Hz, 1H), 6.79–6.65 (m, 2H), 4.25 (t, *J* = 6.9 Hz, 2H), 3.75 (s, 3H), 3.58 (s, 2H), 2.83 (s, 2H), 2.73 (t, *J* = 6.9 Hz, 2H), 2.59 (d, *J* = 11.4 Hz, 2H), 2.53 (s, 3H), 2.43–2.30 (m, 2H), 2.18 (s, 3H), 1.94 (d, *J* = 13.6 Hz, 2H), 1.79–1.68 (m, 2H). LCMS (ESI): *R*_t_ = 1.16 min, *m*/*z* = 601.3 [M + H]^+^.

#### Ethyl (*E*)-5-(3-(2-((8-Methyl-7-(2-methylpyridin-4-yl)-4-oxospiro­[chromane-2,4’-piperidin]-1’-yl)­methyl)­pyridin-3-yl)­propanamido)­pent-2-enoate (22)

^1^H NMR (400 MHz, DMSO) δ 8.53 (d, *J* = 5.0 Hz, 1H), 8.29 (dd, *J* = 4.8, 1.7 Hz, 1H), 7.91 (t, *J* = 5.7 Hz, 1H), 7.64 (d, *J* = 8.1 Hz, 1H), 7.56 (dd, *J* = 7.7, 1.7 Hz, 1H), 7.27 (d, *J* = 1.6 Hz, 1H), 7.26–7.17 (m, 2H), 6.91 (d, *J* = 8.1 Hz, 1H), 6.82 (dt, *J* = 15.7, 6.9 Hz, 1H), 5.85 (dt, *J* = 15.7, 1.6 Hz, 1H), 4.09 (q, *J* = 7.1 Hz, 2H), 3.65 (s, 2H), 3.18 (q, *J* = 6.4 Hz, 2H), 2.95 (dd, *J* = 8.9, 6.6 Hz, 2H), 2.81 (s, 2H), 2.61 (s, 2H), 2.54 (s, 3H), 2.46–2.37 (m, 4H), 2.31 (qd, *J* = 6.6, 1.6 Hz, 2H), 2.18 (s, 3H), 1.92 (d, *J* = 13.6 Hz, 2H), 1.67 (td, *J* = 13.0, 4.3 Hz, 2H), 1.19 (t, *J* = 7.1 Hz, 3H). LCMS (ESI): *R*_t_ = 1.29 min, *m*/*z* = 611.1 [M + H]^+^.

#### Ethyl (*E*)-4-((4-(2-((8-Methyl-7-(2-methylpyridin-4-yl)-4-oxospiro­[chromane-2,4’-piperidin]-1’-yl)­methyl)­pyridin-3-yl)­butyl)­amino)-4-oxobut-2-enoate (23)

^1^H NMR (400 MHz, DMSO) δ 8.53 (t, *J* = 4.4 Hz, 2H), 8.29 (dd, *J* = 4.8, 1.7 Hz, 1H), 7.64 (d, *J* = 8.1 Hz, 1H), 7.59 (dd, *J* = 7.7, 1.7 Hz, 1H), 7.27 (s, 1H), 7.26–7.17 (m, 2H), 6.99 (d, *J* = 15.5 Hz, 1H), 6.91 (d, *J* = 8.1 Hz, 1H), 6.56 (d, *J* = 15.5 Hz, 1H), 4.18 (q, *J* = 7.1 Hz, 2H), 3.64 (s, 2H), 3.21 (t, *J* = 6.3 Hz, 2H), 2.81 (s, 2H), 2.72 (t, *J* = 7.6 Hz, 2H), 2.60 (s, 2H), 2.54 (s, 3H), 2.46–2.38 (m, 2H), 2.18 (s, 3H), 1.92 (d, *J* = 13.5 Hz, 2H), 1.63 (q, *J* = 13.2 Hz, 4H), 1.53 (q, *J* = 7.1 Hz, 2H), 1.24 (d, *J* = 7.1 Hz, 3H). LCMS (ESI): *R*_t_ = 1.38 min, *m*/*z* = 611.2 [M + H]^+^.

#### *N*’-(3-(2-((8-Methyl-7-(2-methylpyridin-4-yl)-4-oxospiro­[chromane-2,4’-piperidin]-1’-yl)­methyl)­pyridin-3-yl)­propanoyl)­acrylohydrazide (24)

^1^H NMR (400 MHz, DMSO) δ 10.02 (s, 2H), 8.54–8.48 (m, 1H), 8.29 (dd, *J* = 4.8, 1.7 Hz, 1H), 7.66–7.58 (m, 2H), 7.27–7.12 (m, 3H), 6.89 (d, *J* = 8.1 Hz, 1H), 6.36–6.13 (m, 2H), 5.69 (dd, *J* = 10.0, 2.4 Hz, 1H), 3.66 (s, 2H), 2.97 (t, *J* = 7.8 Hz, 2H), 2.81 (s, 2H), 2.58 (d, *J* = 12.6 Hz, 4H), 2.52 (s, 3H), 2.45–2.35 (m, 2H), 2.17 (s, 3H), 1.90 (d, *J* = 13.5 Hz, 2H), 1.70 (td, *J* = 13.1, 4.3 Hz, 2H). LCMS (ESI): *R*_t_ = 1.14 min, *m*/*z* = 554.2 [M + H]^+^.

#### (*E*)-*N*’-(3-(2-((8-Methyl-7-(2-methylpyridin-4-yl)-4-oxospiro­[chromane-2,4’-piperidin]-1’-yl)­methyl)­pyridin-3-yl)­propanoyl)-4-oxopent-2-enehydrazide (25)

^1^H NMR (400 MHz, DMSO) δ 10.53 (s, 1H), 10.19 (s, 1H), 8.53 (d, *J* = 5.1 Hz, 1H), 8.30 (dd, *J* = 4.9, 1.7 Hz, 1H), 7.68–7.55 (m, 2H), 7.29–7.19 (m, 2H), 6.97–6.82 (m, 2H), 3.67 (s, 2H), 2.98 (d, *J* = 8.2 Hz, 2H), 2.82 (d, *J* = 6.8 Hz, 2H), 2.59 (d, *J* = 7.1 Hz, 4H), 2.54 (s, 3H), 2.41 (d, *J* = 11.1 Hz, 2H), 2.33 (s, 3H), 2.19 (s, 3H), 1.92 (d, *J* = 13.5 Hz, 2H), 1.70 (d, *J* = 12.7 Hz, 2H). LCMS (ESI): *R*_t_ = 1.13 min, *m*/*z* = 596.2 [M + H]^+^.

#### (*E*)-4-(2-(3-(2-((8-Methyl-7-(2-methylpyridin-4-yl)-4-oxospiro­[chromane-2,4’-piperidin]-1’-yl)­methyl)­pyridin-3-yl)­propanoyl)­hydrazineyl)-4-oxobut-2-enoic Acid (26)

^1^H NMR (400 MHz, DMSO) δ 10.51 (s, 1H), 10.21 (s, 1H), 8.54 (d, *J* = 5.1 Hz, 1H), 8.40 (s, 1H), 7.71 (s, 1H), 7.66 (d, *J* = 8.1 Hz, 1H), 7.32 (s, 1H), 7.26 (s, 1H), 7.24–7.15 (m, 1H), 7.02–6.87 (m, 2H), 6.59 (d, *J* = 15.5 Hz, 1H), 4.04 (s, 2H), 2.95 (d, *J* = 7.5 Hz, 2H), 2.89 (s, 2H), 2.58 (d, *J* = 7.7 Hz, 4H), 2.54 (s, 3H), 2.33 (t, *J* = 1.9 Hz, 2H), 2.20 (s, 3H), 2.05 (s, 2H), 1.91 (s, 2H). LCMS (ESI): *R*_t_ = 1.09 min, *m*/*z* = 598.0 [M + H]^+^.

#### Ethyl (*E*)-4-(2-(3-(2-((8-Methyl-7-(2-methylpyridin-4-yl)-4-oxospiro­[chromane-2,4’-piperidin]-1’-yl)­methyl)­pyridin-3-yl)­propanoyl)­hydrazineyl)-4-oxobut-2-enoate (27)

^1^H NMR (400 MHz, DMSO) δ 10.59–10.48 (m, 1H), 10.21 (s, 1H), 8.54 (d, *J* = 5.1 Hz, 1H), 8.40 (s, 1H), 7.68 (dd, *J* = 18.2, 7.6 Hz, 2H), 7.42–7.28 (m, 2H), 7.26 (s, 1H), 7.19 (d, *J* = 5.2 Hz, 1H), 7.08–6.96 (m, 2H), 6.93 (d, *J* = 8.1 Hz, 1H), 6.64 (d, *J* = 15.5 Hz, 1H), 4.19 (q, *J* = 7.1 Hz, 2H), 3.64 (s, 2H), 2.97 (d, *J* = 7.4 Hz, 2H), 2.89 (s, 2H), 2.58 (t, *J* = 8.4 Hz, 3H), 2.54 (s, 2H), 2.51 (d, *J* = 7.4 Hz, 2H), 2.20 (s, 3H), 2.10–1.75 (m, 4H), 1.24 (t, *J* = 7.1 Hz, 3H). LCMS (ESI): *R*_t_ = 1.20 min, *m*/*z* = 626.1 [M + H]^+^.

#### (*E*)-*N*-Methyl-4-(2-(3-(2-((8-methyl-7-(2-methylpyridin-4-yl)-4-oxospiro­[chromane-2,4’-piperidin]-1’-yl)­methyl)­pyridin-3-yl)­propanoyl)­hydrazineyl)-4-oxobut-2-enamide (28)

General Procedure H was followed using **26** (0.10 g, 0.17 mmol, 1.0 equiv) to obtain the title compound (22.0 mg, 22%). ^1^H NMR (400 MHz, DMSO) δ 10.36 (s, 1H), 10.05 (s, 1H), 8.53 (d, *J* = 5.1 Hz, 1H), 8.39 (d, *J* = 4.9 Hz, 1H), 8.30 (dd, *J* = 4.8, 1.7 Hz, 1H), 7.67–7.61 (m, 2H), 7.27 (s, 1H), 7.26–7.17 (m, 2H), 6.94–6.85 (m, 3H), 3.67 (s, 2H), 2.99 (t, *J* = 7.8 Hz, 2H), 2.82 (s, 2H), 2.70 (d, *J* = 4.6 Hz, 3H), 2.60 (d, *J* = 12.3 Hz, 4H), 2.54 (s, 3H), 2.42 (t, *J* = 11.3 Hz, 2H), 2.19 (s, 3H), 1.92 (d, *J* = 13.5 Hz, 2H), 1.72 (q, *J* = 8.4 Hz, 2H). LCMS (ESI): *R*_t_ = 1.08 min, *m*/*z* = 611.2 [M + H]^+^.

#### (*E*)-*N*,*N*-Dimethyl-4-(2-(3-(2-((8-methyl-7-(2-methylpyridin-4-yl)-4-oxospiro­[chromane-2,4’-piperidin]-1’-yl)­methyl)­pyridin-3-yl)­propanoyl)­hydrazineyl)-4-oxobut-2-enamide (29)

General Procedure H was followed using **26** (0.10 g, 0.17 mmol, 1.0 equiv) to obtain the title compound (20.0 mg, 19%).^1^H NMR (400 MHz, DMSO) δ 10.38 (s, 1H), 10.07 (s, 1H), 8.53 (d, *J* = 5.1 Hz, 1H), 8.30 (dd, *J* = 4.8, 1.7 Hz, 1H), 7.68–7.58 (m, 2H), 7.33 (d, *J* = 15.1 Hz, 1H), 7.27 (d, *J* = 1.7 Hz, 1H), 7.26–7.17 (m, 2H), 6.95–6.81 (m, 2H), 3.67 (s, 2H), 3.09 (s, 3H), 2.99 (t, *J* = 7.7 Hz, 2H), 2.92 (s, 3H), 2.83 (s, 2H), 2.61 (s, 4H), 2.54 (s, 3H), 2.46–2.36 (m, 2H), 2.19 (s, 3H), 1.92 (d, *J* = 13.4 Hz, 2H), 1.77–1.66 (m, 2H). LCMS (ESI): *R*_t_ = 1.10 min, *m*/*z* = 625.3 [M + H]^+^.

#### (*E*)-*N*’-(3-(2-((8-Methyl-7-(2-methylpyridin-4-yl)-4-oxospiro­[chromane-2,4’-piperidin]-1’-yl)­methyl)­pyridin-3-yl)­propanoyl)-4-morpholino-4-oxobut-2-enehydrazide (30)

General Procedure H was followed using **26** (0.10 g, 0.17 mmol, 1.0 equiv) to obtain the title compound (28.0 mg, 25%). ^1^H NMR (400 MHz, DMSO) δ 10.40 (s, 1H), 10.08 (s, 1H), 8.53 (d, *J* = 5.1 Hz, 1H), 8.30 (dd, *J* = 4.8, 1.7 Hz, 1H), 7.68–7.56 (m, 2H), 7.35 (d, *J* = 15.1 Hz, 1H), 7.30–7.15 (m, 3H), 6.96–6.83 (m, 2H), 3.67 (s, 2H), 3.57 (d, *J* = 19.2 Hz, 8H), 2.99 (t, *J* = 7.8 Hz, 2H), 2.83 (s, 2H), 2.59 (q, *J* = 8.0 Hz, 4H), 2.54 (s, 3H), 2.42 (t, *J* = 11.2 Hz, 2H), 2.19 (s, 3H), 1.92 (d, *J* = 13.5 Hz, 2H), 1.71 (td, *J* = 13.0, 4.3 Hz, 2H). LCMS (ESI): *R*_t_ = 1.12 min, *m*/*z* = 667.3 [M + H]^+^.

#### (*E*)-*N*’-(3-(2-((8-Methyl-7-(2-methylpyridin-4-yl)-4-oxospiro­[chromane-2,4’-piperidin]-1’-yl)­methyl)­pyridin-3-yl)­propanoyl)-4-oxo-4-(piperazin-1-yl)­but-2-enehydrazide (31)

General Procedure H was followed using **26** (0.10 g, 0.17 mmol, 1.0 equiv) to obtain the title compound (10.0 mg, 9%). ^1^H NMR (400 MHz, DMSO) δ 10.39 (s, 1H), 10.08 (s, 1H), 8.53 (d, *J* = 5.1 Hz, 1H), 8.30 (dd, *J* = 4.8, 1.7 Hz, 1H), 7.70–7.60 (m, 2H), 7.34 (d, *J* = 15.1 Hz, 1H), 7.30–7.26 (m, 1H), 7.26–7.17 (m, 2H), 6.91 (d, *J* = 8.1 Hz, 1H), 6.85 (d, *J* = 15.1 Hz, 1H), 3.67 (s, 2H), 3.53 (q, *J* = 4.7 Hz, 4H), 2.99 (t, *J* = 7.8 Hz, 2H), 2.83 (s, 2H), 2.73–2.65 (m, 1H), 2.64–2.55 (m, 4H), 2.54 (s, 3H), 2.47–2.35 (m, 6H), 2.19 (s, 3H), 1.92 (d, *J* = 13.5 Hz, 2H), 1.80–1.62 (m, 2H), 0.97 (d, *J* = 6.5 Hz, 6H). LCMS (ESI): *R*_t_ = 1.09 min, *m*/*z* = 666.3 [M + H]^+^.

#### (*E*)-4-(4-Isopropylpiperazin-1-yl)-*N*’-(3-(2-((8-methyl-7-(2-methylpyridin-4-yl)-4-oxospiro­[chromane-2,4’-piperidin]-1’-yl)­methyl)­pyridin-3-yl)­propanoyl)-4-oxobut-2-enehydrazide (32)

General Procedure H was followed using **26** (0.10 g, 0.17 mmol, 1.0 equiv) to obtain the title compound (19.0 mg, 16%). ^1^H NMR (400 MHz, DMSO) δ 10.39 (s, 1H), 10.08 (s, 1H), 8.53 (d, *J* = 5.1 Hz, 1H), 8.30 (dd, *J* = 4.8, 1.7 Hz, 1H), 7.70–7.60 (m, 2H), 7.34 (d, *J* = 15.1 Hz, 1H), 7.30–7.26 (m, 1H), 7.26–7.17 (m, 2H), 6.91 (d, *J* = 8.1 Hz, 1H), 6.85 (d, *J* = 15.1 Hz, 1H), 3.67 (s, 2H), 3.53 (q, *J* = 4.7 Hz, 4H), 2.99 (t, *J* = 7.8 Hz, 2H), 2.83 (s, 2H), 2.73–2.65 (m, 1H), 2.64–2.55 (m, 4H), 2.54 (s, 3H), 2.47–2.35 (m, 6H), 2.19 (s, 3H), 1.92 (d, *J* = 13.5 Hz, 2H), 1.80–1.62 (m, 2H), 0.97 (d, *J* = 6.5 Hz, 6H). LCMS (ESI): *R*_t_ = 1.09 min, *m*/*z* = 708.9 [M + H]^+^.

#### (*E*)-4-(3-(Dimethylamino)­azetidin-1-yl)-*N*’-(3-(2-((8-methyl-7-(2-methylpyridin-4-yl)-4-oxospiro­[chromane-2,4’-piperidin]-1’-yl)­methyl)­pyridin-3-yl)­propanoyl)-4-oxobut-2-enehydrazide (33)

General Procedure H was followed using **26** (0.10 g, 0.17 mmol, 1.0 equiv) to obtain the title compound (20.0 mg, 18%). ^1^H NMR (400 MHz, DMSO) δ 10.42 (s, 1H), 10.09 (s, 1H), 8.53 (d, *J* = 5.1 Hz, 1H), 8.30 (dd, *J* = 4.8, 1.7 Hz, 1H), 7.67–7.58 (m, 2H), 7.31–7.17 (m, 3H), 6.90 (d, *J* = 13.6 Hz, 3H), 4.33–4.25 (m, 1H), 4.08 (dd, *J* = 9.1, 5.0 Hz, 1H), 3.96 (dd, *J* = 10.3, 7.5 Hz, 1H), 3.73 (dd, *J* = 10.6, 5.1 Hz, 1H), 3.67 (s, 2H), 3.08 (tt, *J* = 7.2, 5.0 Hz, 1H), 2.99 (t, *J* = 7.8 Hz, 2H), 2.82 (s, 2H), 2.58 (q, *J* = 8.3 Hz, 4H), 2.54 (s, 3H), 2.42 (t, *J* = 11.3 Hz, 2H), 2.19 (s, 3H), 2.08 (d, *J* = 6.4 Hz, 6H), 1.92 (d, *J* = 13.4 Hz, 2H), 1.71 (td, *J* = 15.0, 4.2 Hz, 2H). LCMS (ESI): *R*_t_ = 1.10 min, *m*/*z* = 680.4 [M + H]^+^.

#### (*E*)-4-(3,3-Difluoroazetidin-1-yl)-*N*’-(3-(2-((8-methyl-7-(2-methylpyridin-4-yl)-4-oxospiro­[chromane-2,4’-piperidin]-1’-yl)­methyl)­pyridin-3-yl)­propanoyl)-4-oxobut-2-enehydrazide (34)

General Procedure H was followed using **26** (0.10 g, 0.17 mmol, 1.0 equiv) to obtain the title compound (29.0 mg, 26%). ^1^H NMR (400 MHz, DMF) δ 10.89 (s, 1H), 10.55 (s, 1H), 8.95 (d, *J* = 5.1 Hz, 1H), 8.72 (dd, *J* = 4.8, 1.8 Hz, 1H), 8.10–8.00 (m, 2H), 7.72–7.58 (m, 3H), 7.40–7.19 (m, 3H), 5.23 (t, *J* = 12.4 Hz, 2H), 4.82 (t, *J* = 12.5 Hz, 2H), 4.09 (s, 2H), 3.41 (t, *J* = 7.7 Hz, 2H), 3.24 (s, 2H), 3.02 (d, *J* = 7.6 Hz, 4H), 2.96 (s, 3H), 2.84 (t, *J* = 11.3 Hz, 2H), 2.61 (d, *J* = 2.7 Hz, 3H), 2.34 (d, *J* = 13.6 Hz, 2H), 2.19–2.06 (m, 2H). LCMS (ESI): *R*_t_ = 1.20 min, *m*/*z* = 673.3 [M + H]^+^.

#### (*E*)-*N*’-(3-(2-((8-Methyl-7-(2-methylpyridin-4-yl)-4-oxospiro­[chromane-2,4’-piperidin]-1’-yl)­methyl)­pyridin-3-yl)­propanoyl)-4-oxo-4-(2-oxa-6-azaspiro­[3.3]­heptan-6-yl)­but-2-enehydrazide (35)

General Procedure H was followed using **26** (0.10 g, 0.17 mmol, 1.0 equiv) to obtain the title compound (23.0 mg, 20%). ^1^H NMR (400 MHz, DMSO) δ 10.42 (s, 1H), 10.09 (s, 1H), 8.53 (d, *J* = 5.1 Hz, 1H), 8.30 (dd, *J* = 4.8, 1.7 Hz, 1H), 7.69–7.58 (m, 2H), 7.28–7.17 (m, 3H), 6.93–6.82 (m, 3H), 4.68 (s, 4H), 4.47 (s, 2H), 4.13 (s, 2H), 3.67 (s, 2H), 2.99 (t, *J* = 7.7 Hz, 2H), 2.82 (s, 2H), 2.64–2.55 (m, 4H), 2.53 (s, 3H), 2.47–2.37 (m, 2H), 2.19 (s, 3H), 1.92 (d, *J* = 13.4 Hz, 2H), 1.71 (td, *J* = 13.2, 4.3 Hz, 2H). LCMS (ESI): *R*_t_ = 1.12 min, *m*/*z* = 679.3 [M + H]^+^.

#### (*E*)-*N*’-(3-(2-((8-Methyl-7-(2-methylpyridin-4-yl)-4-oxospiro­[chromane-2,4’-piperidin]-1’-yl)­methyl)­pyridin-3-yl)­propanoyl)-4-(6-methyl-2,6-diazaspiro­[3.3]­heptan-2-yl)-4-oxobut-2-enehydrazide (36)

General Procedure H was followed using **26** (0.10 g, 0.17 mmol, 1.0 equiv) to obtain the title compound (16.0 mg, 14%). ^1^H NMR (400 MHz, DMSO) δ 10.40 (s, 1H), 10.08 (s, 1H), 8.53 (d, *J* = 5.1 Hz, 1H), 8.30 (dd, *J* = 4.8, 1.7 Hz, 1H), 7.64 (t, *J* = 8.0 Hz, 2H), 7.30–7.16 (m, 3H), 6.94–6.81 (m, 3H), 4.35 (s, 2H), 4.01 (s, 2H), 3.67 (s, 2H), 3.29 (s, 4H), 2.98 (t, *J* = 7.7 Hz, 2H), 2.82 (s, 2H), 2.58 (d, *J* = 7.5 Hz, 4H), 2.54 (s, 3H), 2.42 (t, *J* = 11.3 Hz, 2H), 2.20 (d, *J* = 4.0 Hz, 6H), 1.92 (d, *J* = 13.4 Hz, 2H), 1.72 (d, *J* = 12.8 Hz, 2H). LCMS (ESI): *R*_t_ = 1.10 min, *m*/*z* = 692.4 [M + H]^+^.

#### (*E*)-4-(7,8-Dihydropyrido­[4,3-*d*]­pyrimidin-6­(5*H*)-yl)-*N*’-(3-(2-((8-methyl-7-(2-methylpyridin-4-yl)-4-oxospiro­[chromane-2,4’-piperidin]-1’-yl)­methyl)­pyridin-3-yl)­propanoyl)-4-oxobut-2-enehydrazide (37)

General Procedure H was followed using **26** (0.10 g, 0.17 mmol, 1.0 equiv) to obtain the title compound (32.0 mg, 27%). ^1^H NMR (400 MHz, DMSO) δ 10.43 (s, 1H), 10.10 (s, 1H), 8.97 (s, 1H), 8.68 (d, *J* = 6.1 Hz, 1H), 8.53 (d, *J* = 5.1 Hz, 1H), 8.31 (dd, *J* = 4.8, 1.7 Hz, 1H), 7.67–7.59 (m, 2H), 7.47 (d, *J* = 15.1 Hz, 1H), 7.30–7.17 (m, 3H), 6.93 (dd, *J* = 13.6, 7.5 Hz, 2H), 4.91 (s, 1H), 4.78 (s, 1H), 3.92 (dd, *J* = 12.0, 6.2 Hz, 2H), 3.68 (s, 2H), 3.02–2.88 (m, 4H), 2.83 (s, 2H), 2.67–2.57 (m, 4H), 2.54 (s, 3H), 2.41 (d, *J* = 11.1 Hz, 2H), 2.19 (s, 3H), 1.93 (d, *J* = 13.5 Hz, 2H), 1.73 (d, *J* = 13.9 Hz, 2H). LCMS (ESI): *R*_t_ = 1.13 min, *m*/*z* = 715.3 [M + H]^+^.

#### (*E*)-4-(2,3-Dihydro-1*H*-pyrido­[3,4-*b*]­[1,4]­oxazin-1-yl)-*N*’-(3-(2-((8-methyl-7-(2-methylpyridin-4-yl)-4-oxospiro­[chromane-2,4’-piperidin]-1’-yl)­methyl)­pyridin-3-yl)­propanoyl)-4-oxobut-2-enehydrazide (38)

General Procedure H was followed using **26** (0.10 g, 0.17 mmol, 1.0 equiv) to obtain the title compound (20.0 mg, 17%). ^1^H NMR (400 MHz, DMSO) δ 10.55 (s, 1H), 10.17 (s, 1H), 8.53 (d, *J* = 5.0 Hz, 1H), 8.30 (dd, *J* = 4.8, 1.7 Hz, 1H), 8.22 (s, 1H), 8.06 (d, *J* = 5.5 Hz, 1H), 7.80–7.59 (m, 3H), 7.35 (d, *J* = 15.1 Hz, 1H), 7.30–7.17 (m, 3H), 7.03 (d, *J* = 15.0 Hz, 1H), 6.91 (d, *J* = 8.1 Hz, 1H), 4.41–4.31 (m, 2H), 4.00 (t, *J* = 4.6 Hz, 2H), 3.68 (s, 2H), 3.00 (t, *J* = 7.8 Hz, 2H), 2.83 (s, 2H), 2.65–2.55 (m, 4H), 2.54 (s, 3H), 2.42 (t, *J* = 11.3 Hz, 2H), 2.18 (d, *J* = 5.3 Hz, 3H), 1.92 (d, *J* = 13.4 Hz, 2H), 1.78–1.65 (m, 2H). LCMS (ESI): *R*_t_ = 1.13 min, *m*/*z* = 716.4 [M + H]^+^.

#### Methyl 3-(2-((8-Methyl-7-(2-methylpyridin-4-yl)­spiro­[chromane-2,4’-piperidin]-1’-yl)­methyl)­pyridin-3-yl)­propanoate (I-39)

General Procedure F were followed sequentially using **I-7** (6.0 g, 19.0 mmol, 1.0 equiv) and **I-24** (4.6 g, 21.0 mmol, 1.1 equiv) to obtain the title compound (8.0 g, 85%). LCMS (ESI): *R*_t_ = 1.32 min, *m*/*z* = 486.2 [M + H]^+^.

#### 3-(2-((8-Methyl-7-(2-methylpyridin-4-yl)­spiro­[chromane-2,4’-piperidin]-1’-yl)­methyl)­pyridin-3-yl)­propanehydrazide (I-40)

General Procedure G was followed using **I-39** (4.5 g, 9.27 mmol, 1.0 equiv) to obtain the title compound (4.20 g, 95%). LCMS (ESI): Rt = 1.22 min, *m*/*z* = 472.2 [M + H]^+^.

#### (*E*)-4-(2-(3-(2-((8-Methyl-7-(2-methylpyridin-4-yl)­spiro­[chromane-2,4’-piperidin]-1’-yl)­methyl)­pyridin-3-yl)­propanoyl)­hydrazineyl)-4-oxobut-2-enoic Acid (I-41)

General Procedures H and G were followed sequentially using **I-40** (4.20 g, 8.91 mmol, 1.0 equiv) to obtain the title compound (3.70 g, 72%). LCMS (ESI): *R*_t_ = 1.23 min, *m*/*z* = 584.2 [M + H]^+^.

#### (*E*)-*N*’-(3-(2-((8-Methyl-7-(2-methylpyridin-4-yl)­spiro­[chromane-2,4’-piperidin]-1’-yl)­methyl)­pyridin-3-yl)­propanoyl)-4-(4-methylpiperazin-1-yl)-4-oxobut-2-enehydrazide (39)

General Procedure H was followed using **I-41** (0.10 g, 0.17 mmol, 1.0 equiv) to obtain the title compound (33.0 mg, 29%). ^1^H NMR (400 MHz, DMSO) δ 10.41 (s, 1H), 10.12 (s, 1H), 8.46 (d, *J* = 5.1 Hz, 1H), 8.31 (dd, *J* = 4.9, 1.7 Hz, 1H), 7.64 (dd, *J* = 7.7, 1.7 Hz, 1H), 7.35 (d, *J* = 15.2 Hz, 1H), 7.23 (dd, *J* = 7.7, 4.7 Hz, 1H), 7.19 (s, 1H), 7.12 (dd, *J* = 5.1, 1.6 Hz, 1H), 6.98 (d, *J* = 7.8 Hz, 1H), 6.86 (d, *J* = 15.1 Hz, 1H), 6.67 (d, *J* = 7.8 Hz, 1H), 3.68 (s, 2H), 3.54 (p, *J* = 3.1 Hz, 4H), 3.02 (t, *J* = 7.6 Hz, 2H), 2.75 (t, *J* = 6.7 Hz, 2H), 2.59 (t, *J* = 7.7 Hz, 4H), 2.50 (d, *J* = 2.9 Hz, 3H), 2.44 (t, *J* = 12.5 Hz, 2H), 2.30 (dt, *J* = 10.1, 4.8 Hz, 4H), 2.19 (s, 3H), 2.11 (s, 3H), 1.77 (t, *J* = 6.7 Hz, 2H), 1.70 (d, *J* = 13.2 Hz, 2H), 1.66–1.55 (m, 2H). LCMS (ESI): *R*_t_ = 1.22 min, *m*/*z* = 666.4 [M + H]^+^.

#### (*E*)-4-(4-Isopropylpiperazin-1-yl)-*N*’-(3-(2-((8-methyl-7-(2-methylpyridin-4-yl)­spiro­[chromane-2,4’-piperidin]-1’-yl)­methyl)­pyridin-3-yl)­propanoyl)-4-oxobut-2-enehydrazide (40)

General Procedure H was followed using **I-41** (0.10 g, 0.17 mmol, 1.0 equiv) to obtain the title compound (23.0 mg, 19%). ^1^H NMR (400 MHz, DMSO) δ 10.52 (s, 1H), 9.93 (s, 1H), 8.53 (t, *J* = 5.1 Hz, 1H), 8.37 (s, 1H), 7.75–7.62 (m, 1H), 7.34–7.13 (m, 3H), 7.11–6.98 (m, 1H), 6.80 (d, *J* = 7.7 Hz, 1H), 6.74 (d, *J* = 7.8 Hz, 1H), 6.53 (d, *J* = 9.8 Hz, 1H), 3.74 (s, 2H), 3.05 (d, *J* = 6.8 Hz, 2H), 2.86–2.69 (m, 5H), 2.63 (d, *J* = 7.1 Hz, 4H), 2.57 (s, 3H), 2.44 (d, *J* = 40.5 Hz, 8H), 2.17 (s, 3H), 2.00–1.45 (m, 6H), 1.09–0.84 (m, 6H). LCMS (ESI): *R*_t_ = 1.22 min, *m*/*z* = 694.4 [M + H]^+^.

#### (*E*)-*N*’-(3-(2-((8-Methyl-7-(2-methylpyridin-4-yl)­spiro­[chromane-2,4’-piperidin]-1’-yl)­methyl)­pyridin-3-yl)­propanoyl)-4-oxo-4-(2-oxa-6-azaspiro­[3.3]­heptan-6-yl)­but-2-enehydrazide (41)

General Procedure H was followed using **I-41** (0.10 g, 0.17 mmol, 1.0 equiv) to obtain the title compound (40.0 mg, 35%). ^1^H NMR (400 MHz, DMSO) δ 10.42 (s, 1H), 10.12 (s, 1H), 8.46 (d, *J* = 5.1 Hz, 1H), 8.31 (d, *J* = 4.8 Hz, 1H), 7.69–7.60 (m, 1H), 7.24 (dd, *J* = 7.7, 4.8 Hz, 1H), 7.19 (d, *J* = 1.6 Hz, 1H), 7.12 (dd, *J* = 5.1, 1.7 Hz, 1H), 6.99 (d, *J* = 7.8 Hz, 1H), 6.91–6.80 (m, 2H), 6.68 (d, *J* = 7.8 Hz, 1H), 4.67 (s, 4H), 4.46 (s, 2H), 4.12 (s, 2H), 3.67 (s, 2H), 3.01 (t, *J* = 7.6 Hz, 2H), 2.75 (t, *J* = 6.7 Hz, 2H), 2.58 (t, *J* = 7.8 Hz, 4H), 2.51 (s, 3H), 2.44 (s, 2H), 2.11 (s, 3H), 1.77 (t, *J* = 6.7 Hz, 2H), 1.70 (d, *J* = 13.0 Hz, 2H), 1.63 (d, *J* = 13.5 Hz, 2H). LCMS (ESI): *R*_t_ = 1.23 min, *m*/*z* = 665.4 [M + H]^+^.

#### (*E*)-4-(3-(Dimethylamino)­azetidin-1-yl)-*N*’-(3-(2-((8-methyl-7-(2-methylpyridin-4-yl)­spiro­[chromane-2,4’-piperidin]-1’-yl)­methyl)­pyridin-3-yl)­propanoyl)-4-oxobut-2-enehydrazide (42)

General Procedure H was followed using **I-41** (0.10 g, 0.17 mmol, 1.0 equiv) to obtain the title compound (17.0 mg, 15%). ^1^H NMR (400 MHz, DMSO) δ 10.43 (s, 1H), 10.13 (s, 1H), 8.46 (d, *J* = 5.1 Hz, 1H), 8.31 (dd, *J* = 4.7, 1.6 Hz, 1H), 7.64 (dd, *J* = 7.7, 1.7 Hz, 1H), 7.24 (dd, *J* = 7.7, 4.8 Hz, 1H), 7.19 (d, *J* = 1.6 Hz, 1H), 7.12 (dd, *J* = 5.1, 1.7 Hz, 1H), 6.98 (d, *J* = 7.8 Hz, 1H), 6.88 (s, 2H), 6.68 (d, *J* = 7.8 Hz, 1H), 4.29 (t, *J* = 8.1 Hz, 1H), 4.07 (dd, *J* = 9.0, 5.0 Hz, 1H), 3.95 (dd, *J* = 10.6, 7.2 Hz, 1H), 3.77–3.61 (m, 3H), 3.08 (tt, *J* = 7.2, 5.1 Hz, 1H), 3.01 (t, *J* = 7.7 Hz, 2H), 2.75 (t, *J* = 6.8 Hz, 2H), 2.59 (d, *J* = 7.7 Hz, 4H), 2.51 (s, 3H), 2.44 (s, 2H), 2.15–2.07 (m, 9H), 1.77 (t, *J* = 6.7 Hz, 2H), 1.70 (d, *J* = 13.2 Hz, 2H), 1.63 (d, *J* = 11.9 Hz, 2H). LCMS (ESI): *R*_t_ = 1.20 min, *m*/*z* = 666.4 [M + H]^+^.

#### (*E*)-*N*’-(3-(2-((8-Methyl-7-(2-methylpyridin-4-yl)­spiro­[chromane-2,4’-piperidin]-1’-yl)­methyl)­pyridin-3-yl)­propanoyl)-4-(6-methyl-2,6-diazaspiro­[3.3]­heptan-2-yl)-4-oxobut-2-enehydrazide (43)

General Procedure H was followed using **I-41** (0.10 g, 0.17 mmol, 1.0 equiv) to obtain the title compound (13.0 mg, 29%). ^1^H NMR (400 MHz, DMSO) δ 10.42 (s, 1H), 10.11 (s, 1H), 8.46 (d, *J* = 5.1 Hz, 1H), 8.31 (dd, *J* = 4.8, 1.7 Hz, 1H), 7.63 (dd, *J* = 7.6, 1.7 Hz, 1H), 7.23 (dt, *J* = 7.6, 3.8 Hz, 1H), 7.19 (d, *J* = 3.2 Hz, 1H), 7.12 (dd, *J* = 5.0, 1.7 Hz, 1H), 6.99 (d, *J* = 7.9 Hz, 1H), 6.86 (dd, *J* = 13.9, 6.5 Hz, 2H), 6.68 (d, *J* = 7.8 Hz, 1H), 4.37 (s, 2H), 4.03 (s, 2H), 3.68 (s, 2H), 3.46 (s, 2H), 3.06–2.96 (m, 2H), 2.74 (d, *J* = 7.5 Hz, 2H), 2.58 (t, *J* = 7.7 Hz, 4H), 2.51 (s, 3H), 2.44 (s, 2H), 2.31 (s, 2H), 2.11 (s, 3H), 2.09 (s, 3H), 1.77 (t, *J* = 6.7 Hz, 2H), 1.70 (d, *J* = 13.4 Hz, 2H), 1.66–1.58 (m, 2H). LCMS (ESI): *R*_t_ = 1.22 min, *m*/*z* = 678.4 [M + H]^+^.

#### (*E*)-4-(3-Fluoroazetidin-1-yl)-*N*’-(3-(2-((8-methyl-7-(2-methylpyridin-4-yl)­spiro­[chromane-2,4’-piperidin]-1’-yl)­methyl)­pyridin-3-yl)­propanoyl)-4-oxobut-2-enehydrazide (44)

General Procedure H was followed using **I-41** (0.10 g, 0.17 mmol, 1.0 equiv) to obtain the title compound (21.0 mg, 38%). ^1^H NMR (400 MHz, DMSO) δ 10.45 (s, 1H), 10.14 (s, 1H), 8.46 (d, *J* = 5.1 Hz, 1H), 8.30 (dt, *J* = 4.2, 2.1 Hz, 1H), 7.63 (dd, *J* = 7.7, 1.7 Hz, 1H), 7.23 (dd, *J* = 7.7, 4.7 Hz, 1H), 7.19 (d, *J* = 1.6 Hz, 1H), 7.12 (dd, *J* = 5.0, 1.7 Hz, 1H), 6.99 (d, *J* = 7.9 Hz, 1H), 6.94–6.80 (m, 2H), 6.68 (d, *J* = 7.8 Hz, 1H), 5.42 (dtd, *J* = 57.2, 6.1, 3.1 Hz, 1H), 4.62 (ddd, *J* = 20.7, 10.4, 6.1 Hz, 1H), 4.45–4.21 (m, 2H), 4.08–3.91 (m, 1H), 3.66 (d, *J* = 3.3 Hz, 2H), 3.01 (q, *J* = 7.7 Hz, 2H), 2.75 (t, *J* = 6.8 Hz, 2H), 2.58 (t, *J* = 7.7 Hz, 4H), 2.51 (s, 3H), 2.47–2.38 (m, 2H), 2.11 (s, 3H), 1.77 (t, *J* = 6.7 Hz, 2H), 1.70 (d, *J* = 13.4 Hz, 2H), 1.67–1.54 (m, 2H). LCMS (ESI): *R*_t_ = 1.20 min, *m*/*z* = 641.4 [M + H]^+^.

#### (*E*)-4-(3,3-Difluoroazetidin-1-yl)-*N*’-(3-(2-((8-methyl-7-(2-methylpyridin-4-yl)­spiro­[chromane-2,4’-piperidin]-1’-yl)­methyl)­pyridin-3-yl)­propanoyl)-4-oxobut-2-enehydrazide (45)

General Procedure H was followed using **I-41** (0.10 g, 0.17 mmol, 1.0 equiv) to obtain the title compound (35.0 mg, 41%). ^1^H NMR (400 MHz, DMSO) δ 10.48 (s, 1H), 10.16 (s, 1H), 8.46 (d, *J* = 5.1 Hz, 1H), 8.31 (dd, *J* = 4.8, 1.7 Hz, 1H), 7.63 (dd, *J* = 7.7, 1.7 Hz, 1H), 7.24 (dd, *J* = 7.7, 4.7 Hz, 1H), 7.19 (s, 1H), 7.12 (dd, *J* = 5.1, 1.7 Hz, 1H), 7.03–6.95 (m, 1H), 6.96–6.83 (m, 2H), 6.68 (d, *J* = 7.8 Hz, 1H), 4.81 (t, *J* = 12.3 Hz, 2H), 4.40 (t, *J* = 12.5 Hz, 2H), 3.68 (s, 2H), 3.01 (t, *J* = 7.7 Hz, 2H), 2.75 (t, *J* = 6.7 Hz, 2H), 2.59 (d, *J* = 7.9 Hz, 4H), 2.51 (s, 3H), 2.44 (s, 2H), 2.11 (s, 3H), 1.77 (t, *J* = 6.7 Hz, 2H), 1.70 (d, *J* = 13.2 Hz, 2H), 1.68–1.56 (m, 2H). LCMS (ESI): *R*_t_ = 1.29 min, *m*/*z* = 659.4 [M + H]^+^.

#### (*E*)-4-(3-(Difluoromethyl)­azetidin-1-yl)-*N*’-(3-(2-((8-methyl-7-(2-methylpyridin-4-yl)­spiro­[chromane-2,4’-piperidin]-1’-yl)­methyl)­pyridin-3-yl)­propanoyl)-4-oxobut-2-enehydrazide (46)

General Procedure H was followed using **I-41** (0.10 g, 0.17 mmol, 1.0 equiv) to obtain the title compound (13.0 mg, 22%). ^1^H NMR (400 MHz, DMSO) δ 10.42 (s, 1H), 10.14 (s, 1H), 8.46 (d, *J* = 5.1 Hz, 1H), 8.31 (dd, *J* = 4.8, 1.8 Hz, 1H), 7.63 (dt, *J* = 5.8, 2.9 Hz, 1H), 7.23 (dd, *J* = 7.7, 4.7 Hz, 1H), 7.19 (d, *J* = 1.6 Hz, 1H), 7.12 (dd, *J* = 5.1, 1.7 Hz, 1H), 6.98 (d, *J* = 7.8 Hz, 1H), 6.94–6.77 (m, 2H), 6.68 (d, *J* = 7.8 Hz, 1H), 6.35 (td, *J* = 56.3, 4.4 Hz, 1H), 4.42 (t, *J* = 9.0 Hz, 1H), 4.23 (dd, *J* = 9.2, 5.5 Hz, 1H), 4.06 (t, *J* = 9.7 Hz, 1H), 3.88 (dd, *J* = 10.7, 5.6 Hz, 1H), 3.67 (s, 2H), 3.21–3.08 (m, 1H), 3.01 (t, *J* = 7.7 Hz, 2H), 2.75 (t, *J* = 6.7 Hz, 2H), 2.58 (dd, *J* = 9.8, 5.6 Hz, 4H), 2.51 (s, 3H), 2.47–2.38 (m, 2H), 2.11 (s, 3H), 1.77 (t, *J* = 6.8 Hz, 2H), 1.69 (d, *J* = 13.4 Hz, 2H), 1.66–1.55 (m, 2H). LCMS (ESI): *R*_t_ = 1.24 min, *m*/*z* = 673.3 [M + H]^+^.

#### (*S*,*E*)-4-(2-(Difluoromethyl)­azetidin-1-yl)-*N*’-(3-(2-((8-methyl-7-(2-methylpyridin-4-yl)­spiro­[chromane-2,4’-piperidin]-1’-yl)­methyl)­pyridin-3-yl)­propanoyl)-4-oxobut-2-enehydrazide (47)

General Procedure H was followed using **I-41** (0.10 g, 0.17 mmol, 1.0 equiv) to obtain the title compound (6.0 mg, 11%). ^1^H NMR (400 MHz, DMSO) δ 10.52 (s, 1H), 10.21 (s, 1H), 8.52 (d, *J* = 5.1 Hz, 1H), 8.37 (dd, *J* = 4.8, 1.7 Hz, 1H), 7.68 (dt, *J* = 8.8, 2.8 Hz, 1H), 7.29 (dd, *J* = 7.7, 4.7 Hz, 1H), 7.25 (s, 1H), 7.18 (dd, *J* = 5.1, 1.7 Hz, 1H), 7.10–6.79 (m, 3H), 6.74 (d, *J* = 7.8 Hz, 1H), 6.42 (dd, *J* = 56.9, 54.1 Hz, 1H), 5.03 (s, 1H), 4.69 (s, 1H), 4.27 (q, *J* = 6.7 Hz, 1H), 3.97 (dd, *J* = 18.5, 9.2 Hz, 1H), 3.73 (s, 2H), 3.16–2.95 (m, 3H), 2.81 (t, *J* = 6.8 Hz, 2H), 2.65 (q, *J* = 9.0 Hz, 4H), 2.51 (s, 3H), 2.47 (d, *J* = 11.6 Hz, 2H), 2.17 (s, 3H), 1.83 (t, *J* = 6.5 Hz, 2H), 1.76 (d, *J* = 13.6 Hz, 2H), 1.67 (d, *J* = 12.4 Hz, 2H). LCMS (ESI): *R*_t_ = 1.27 min, *m*/*z* = 673.3 [M + H]^+^.

#### (*R*,*E*)-4-(2-(Difluoromethyl)­azetidin-1-yl)-*N*’-(3-(2-((8-methyl-7-(2-methylpyridin-4-yl)­spiro­[chromane-2,4’-piperidin]-1’-yl)­methyl)­pyridin-3-yl)­propanoyl)-4-oxobut-2-enehydrazide (48)

General Procedure H was followed using **I-41** (0.10 g, 0.17 mmol, 1.0 equiv) to obtain the title compound (9.0 mg, 15%). ^1^H NMR (400 MHz, DMSO) δ 10.51 (d, *J* = 22.7 Hz, 1H), 10.19 (d, *J* = 18.4 Hz, 1H), 8.52 (d, *J* = 5.1 Hz, 1H), 8.37 (dd, *J* = 4.8, 1.7 Hz, 1H), 7.69 (dd, *J* = 7.7, 1.7 Hz, 1H), 7.29 (dd, *J* = 7.7, 4.7 Hz, 1H), 7.25 (d, *J* = 1.6 Hz, 1H), 7.18 (dd, *J* = 5.1, 1.7 Hz, 1H), 7.10–6.88 (m, 3H), 6.74 (d, *J* = 7.8 Hz, 1H), 6.61–6.18 (m, 1H), 5.03 (s, 1H), 4.81–4.60 (m, 1H), 4.27 (q, *J* = 6.8 Hz, 1H), 4.05–3.90 (m, 1H), 3.73 (s, 2H), 3.21–2.97 (m, 2H), 2.81 (t, *J* = 6.8 Hz, 2H), 2.64 (t, *J* = 7.8 Hz, 4H), 2.57 (s, 3H), 2.49 (s, 2H), 2.38–2.24 (m, 1H), 2.17 (s, 3H), 1.83 (t, *J* = 6.7 Hz, 2H), 1.76 (d, *J* = 13.4 Hz, 2H), 1.67 (t, *J* = 12.4 Hz, 2H). LCMS (ESI): *R*_t_ = 1.26 min, *m*/*z* = 673.3 [M + H]^+^.

#### (*E*)-*N*’-(3-(2-((8-Methyl-7-(2-methylpyridin-4-yl)­spiro­[chromane-2,4’-piperidin]-1’-yl)­methyl)­pyridin-3-yl)­propanoyl)-4-oxo-4-(3-(trifluoromethyl)­azetidin-1-yl)­but-2-enehydrazide (49)

General Procedure H was followed using **I-41** (0.10 g, 0.17 mmol, 1.0 equiv) to obtain the title compound (6.0 mg, 10%). ^1^H NMR (400 MHz, DMF) δ 10.87 (s, 1H), 10.57 (s, 1H), 8.88 (d, *J* = 5.1 Hz, 1H), 8.73 (dd, *J* = 4.8, 1.7 Hz, 1H), 8.05 (dd, *J* = 7.7, 1.7 Hz, 1H), 7.66 (dd, *J* = 7.7, 4.7 Hz, 1H), 7.61 (d, *J* = 1.6 Hz, 1H), 7.54 (dd, *J* = 5.1, 1.7 Hz, 1H), 7.41 (d, *J* = 7.8 Hz, 1H), 7.39–7.21 (m, 2H), 7.10 (d, *J* = 7.8 Hz, 1H), 4.97 (t, *J* = 9.1 Hz, 1H), 4.78 (dd, *J* = 9.6, 5.4 Hz, 1H), 4.68–4.58 (m, 1H), 4.36 (dd, *J* = 10.8, 5.4 Hz, 1H), 4.09 (s, 3H), 3.44 (t, *J* = 7.7 Hz, 2H), 3.17 (t, *J* = 6.7 Hz, 2H), 3.00 (t, *J* = 7.8 Hz, 4H), 2.93 (s, 3H), 2.89–2.82 (m, 2H), 2.53 (s, 3H), 2.19 (t, *J* = 6.7 Hz, 2H), 2.12 (d, *J* = 13.3 Hz, 2H), 2.02 (q, *J* = 11.1 Hz, 2H). LCMS (ESI): *R*_t_ = 1.29 min, *m*/*z* = 691.3 [M + H]^+^.

#### (*E*)-4-(3-Cyanoazetidin-1-yl)-*N*’-(3-(2-((8-methyl-7-(2-methylpyridin-4-yl)­spiro­[chromane-2,4’-piperidin]-1’-yl)­methyl)­pyridin-3-yl)­propanoyl)-4-oxobut-2-enehydrazide (50)

General Procedure H was followed using **I-41** (0.10 g, 0.17 mmol, 1.0 equiv) to obtain the title compound (12.0 mg, 11%). ^1^H NMR (400 MHz, DMSO) δ 10.45 (s, 1H), 10.14 (s, 1H), 8.46 (d, *J* = 5.1 Hz, 1H), 8.31 (d, *J* = 4.7 Hz, 1H), 7.63 (d, *J* = 7.8 Hz, 1H), 7.27–7.21 (m, 1H), 7.20 (s, 1H), 7.13 (d, *J* = 5.3 Hz, 1H), 6.99 (d, *J* = 7.9 Hz, 1H), 6.86 (q, *J* = 15.2 Hz, 2H), 6.68 (d, *J* = 7.8 Hz, 1H), 4.59–4.52 (m, 1H), 4.24 (t, *J* = 9.7 Hz, 1H), 4.10 (dq, *J* = 10.4, 5.7 Hz, 1H), 3.88–3.79 (m, 1H), 3.67 (s, 2H), 3.01 (t, *J* = 7.6 Hz, 2H), 2.75 (d, *J* = 6.9 Hz, 2H), 2.67 (d, *J* = 2.0 Hz, 1H), 2.58 (t, *J* = 7.7 Hz, 4H), 2.52 (s, 3H), 2.33 (d, *J* = 1.9 Hz, 2H), 2.11 (s, 3H), 1.77 (s, 2H), 1.70 (d, *J* = 13.3 Hz, 2H), 1.62 (d, *J* = 13.3 Hz, 2H). LCMS (ESI): *R*_t_ = 1.19 min, *m*/*z* = 648.3 [M + H]^+^.

### Protein Expression and Purification

The gene containing the ubiquitin-like domain and the catalytic core of SARS-CoV-2 PL^Pro^ (residues 71–315) was synthesized with codon optimization for *Escherichia coli* and cloned in the pET28a­(+) vector by GenScript. We made PL^Pro^ constructs to optimize the protein for NMR-based fragment screen (residues 1–314 with C111S and C270S), reversible X-ray crystallography (residues 1–314 with C111S and C270S), covalent X-ray crystallography (N-term His tag, residues 1–314) and the enzymatic assays (residues 1–315 with C270S) were obtained by site mutagenesis. All PL^Pro^ plasmids were transformed into the BL21­(DE3) strain *E. coli*. The bacteria were cultured in Luria–Bertani broth or M9 minimal media containing ^15^NH_4_Cl supplemented with 50 mg/mL Kanamycin at 37 °C until the optimal density at 600 nm reached 0.8 before inducing protein expression by the addition of 0.1 mM IPTG and 0.1 mM ZnCl_2_ at 18 °C for 20 h. The cell pellet was harvested by centrifugation at 5,000 g for 15 min, resuspended in lysis buffer (50 mM Tris pH 7.0, 500 mM NaCl, 5% glycerol, 10 mM imidazole, 5 mM BME, 0.1% Triton X-100 and 1 mM PMSF), and lysed in APV2000 lab homogenizer (SPX flow). Cell lysate was centrifuged at 15,000 g for 45 min and loaded onto HisTrap FF column (Cytiva). The column was washed with 10 column volumes of Buffer A (50 mM Tris pH 7.0, 500 mM NaCl, 5% glycerol, 10 mM imidazole, 5 mM BME) and eluted with Buffer B (50 mM Tris pH 7.0, 500 mM NaCl, 5% glycerol, 500 mM imidazole, 5 mM BME) using a linear gradient program from 0 to 100% Buffer B over 10 column volumes. To the fractions containing PL^Pro^, Thrombin was added to remove the 6xHis tag and dialyzed against Buffer A without imidazole overnight. Then, Tag-cleaved PL^Pro^ was loaded on a HisTrap column. The flowthrough was concentrated and subjected to HiLoad 26/600 Superdex75 pg (Cytiva) and eluted using Buffer C (20 mM HEPES pH 7.0, 150 mM NaCl, 3 mM DTT) for the NMR-based fragment screen or Buffer D (25 mM Tris pH 7.0, 150 mM NaCl, 3 mM DTT) for X-ray crystallography and the enzymatic assays. Protein concentration was quantified by the Pierce 660 nm assay (ThermoFisher).

### Crystallization and Data Collection

Compound **7**. 12 mg/mL PL^Pro^ in buffer (25 mM Tris pH = 7.0, 50 mM NaCl and 10 mM DTT) was incubated with various ligand (100 mM DMSO stocks, final concentration of 0.5–1 mM) at room temperature for 1 h then centrifuged to remove insoluble precipitate. Hanging drops were set up in a 1:1 ratio of protein+ligand:crystallization solution (50 mM HEPES pH = 7.0, Tryptone 2–4% w/v and 10–16% PEG-3350) and incubated at 18 °C allowing vapor diffusion against the corresponding reservoir solution. Large diamond shaped crystal appeared overnight or within 2 days of setup belonging to space group P 6_5_22. Crystals were cryoprotected in mother liquor supplemented with 20% ethylene glycol or 20% glycerol before being flash frozen in liquid nitrogen

Compound **41**. 16 mg/mL protein stock was diluted 11-fold (45 μL protein into 450 μL buffer (25 mM Tris pH = 7.0, 50 mM NaCl and 10 mM DTT)) and incubated with 1 mM ligand (5 μL of 100 mM stock in DMSO) at room temperature for 1 h (final volume of 500 μL). Protein ligand complex then concentrated to 65 μL and another 0.1 mM ligand added prior to tray set up. Hanging drops were set up in a 1:1 ratio of protein+ligand:crystallization solution (150–300 mM magnesium formate and 12–24% PEG-3350) and incubated at 18 °C allowing vapor diffusion against the corresponding reservoir solution. Large cubic crystals appear 2–3 days after tray setup belonging to space group P1. Crystals were cryoprotected by dragging through paratone oil before being flash cooled in liquid nitrogen.

Data sets were acquired at 100 K on the Berkeley Center for Structural Biology (BSB) 8.2.2 beamline at the ALS using a Pilatus3 2 M detector and Diamond Light Source (DLS) - Macromolecular Crystallography (MX) I04 beamline using a Eiger2 XE 16 M detector for compounds **7** and **41** respectively. Diffraction data were indexed and integrated with XDS or DIALS and scaled with AIMLESS. Phasing was accomplished by molecular replacement with Phaser-MR using the structure of SARS-CoV-2 PL^pro^ with C111S (PDB: 6WRH) as starting model. Ligand models were built by eLBOW and manually added to the corresponding electron density. PL^Pro^-ligand cocrystal structures were determined by several cycles of refinement using Phenix and manual modeling with Coot.

### RLKGG AMC Enzymatic Assay

All compounds were stored in 10 mM stock in DMSO. Dose responses of the compounds were generated using an ECHO 555 Liquid Handler (Labcyte, Inc.) directly onto a 384 well black polystyrene flat bottom plate with a nonbinding surface (Corning p/n 3575). Ten mL of recombinant PL^Pro^ enzyme was added at a concentration of 200 nM, and 10 mL of Ac-RLKGG-AMC substrate was added at a concentration of 60 mM (The final concentrations of enzyme and substrate are 80 nM and 30 mM, respectively in an assay buffer containing 5 mM NaCl, 20 mM Tris HCl pH 7.5, 5 mM DTT, and a DMSO concentration of 5%) The fluorometric measurement of AMC cleavage was measured on a Biotek Citation 3 plate reader with an excitation wavelength of 360 nM and an emission wavelength of 460 nm. Relative fluorescence units were converted to % inhibition relative to the vehicle (positive) control wells. The percent inhibition is plotted against compound concentration to generate an IC_50_ (inhibitor concentration with 50% activity relative to vehicle) by fitting the data to a 4-parameter logistic model using XLFit software (Guildford, Surrey, UK). GRL-0617 served as a PL^Pro^ positive control inhibitor (Selleckchem). Positive control wells contained enzyme, substrate, and vehicle, while negative control wells had substrate and vehicle minus the enzyme. The dose range for the test compounds was 500 mM-0.98 mM or 100 mM-0.026 mM for low and high affinity compounds, respectively. For the 0 min time point, RLKGG substrate was added followed by the addition of enzyme and incubated 10 min prior to fluorometric measurement of AMC cleavage. For the 60 min time point, enzyme was incubated with compound for 60 min followed by addition of substrate for 10 min prior to fluorometric measurement of AMC cleavage.

### ISG-15 AMC Enzymatic Assay

All compounds were stored in 10 mM stock in DMSO. Dose responses of the compounds were generated using an ECHO 555 Liquid Handler (Labcyte, Inc.) directly onto a 384 well black polystyrene flat bottom plate with a nonbinding surface (Corning p/n 3575). Ten μL of recombinant PL^Pro^ was added at a concentration of 6 nM, followed by the addition of 10 μL of ISG-15-AMC it a concentration of 400 nM (The final concentrations of enzyme and substrate are 3 nM and 200 μM, respectively, in a buffer containing 50 mM HEPES, 100 mM NaCl, 0.1% Pluronic F-68, 2.5 mM DTT, with a DMSO concentration of 5%). The fluorometric measurement of AMC cleavage was measured on a Biotek Citation 3 plate reader with an excitation wavelength of 360 nM and an emission wavelength of 460 nm. Relative fluorescence units were plotted against compound concentration to generate an IC_50_ (inhibitor concentration with 50% activity relative to vehicle) by fitting the data to a 4-parameter logistic model using XLFit software (Guildford, Surrey, UK). GRL-0617 served as a PL^Pro^ positive control inhibitor (Selleckchem). Positive control wells contained enzyme, substrate, and vehicle, while negative control wells had substrate and vehicle minus the enzyme. The dose range for the test compounds was 500 mM-0.98 mM or 100 mM-0.026 mM for low and high affinity compounds, respectively. For the 0 min time point, ISG-15 AMC substrate was added followed by the addition of enzyme and incubated 5 min prior to fluorometric measurement of AMC cleavage. For the 60 min time point, enzyme was incubated with compound for 60 min followed by addition of substrate for 5 min prior to fluorometric measurement of AMC cleavage.

### A549 Cellular Antiviral Assay

A549 cells were plated at 2.5 × 10^4^ cells per well in black-walled 96-well plates 24h before infection. Cells were then infected with severe acute respiratory syndrome coronavirus 2 (SARS-CoV-2) infectious clone based on the WA1 strain expressing nanoluciferase in place of the Orf7a accessory protein (SARS-CoV-2 nLuc) (GenBank MT844089) (PMCID: PMC8034761) at an MOI of 0.025 PFU/cell in triplicate, and cells were incubated at 37 °C for 45 min. Inocula were removed and replaced with complete medium containing the indicated compound concentrations. All compounds were prepared in dimethyl sulfoxide (DMSO), and the final concentration of DMSO for all compounds at all dilutions was either 0.1% or 0.5%. Luciferase activity was measured at 48 hpi using Nano-Glo Luciferase Assay reagents (Promega) on a Synergy H1 plate reader (BioTek) according to the manufacturer’s instructions. Parallel A549 cell cultures treated with compound dilutions were incubated at 37 °C, and cell toxicity was determined at 48 hpi using CellTiter-Glo Luminescent Cell Viability reagents (Promega) on a GloMax Discover plate reader (Promega) according to the manufacturer’s instructions.

### Molecular Dynamics Simulations

Conformer pools of PL^Pro^ ligands were generated by Torsional sampling (Monte Carlo Multiple Minimum) using the “Conformational Search” function in MacroModel. An implicit solvent model of water was used along with OPLS4, PRCG, maximum iterations of 5000, and a convergence threshold of 0.001 on “gradient”. All conformers within a 5.00 kcal energy window of the lowest energy structure were evaluated, and the configuration of the ligand in its most stable conformer pool was used for further evaluation in the explicitly solvated-molecular-dynamics simulations. Using the “System Builder” function, representative structures of the most stable conformer of each ligand (phenyl, 2-pyridyl, and 3-pyridyl) were prepared for molecular dynamics simulation. The TIP4P solvent model was chosen because of its precedented compatibility with OPLS force fields. The force field was set to OPLS5 because of its greater accuracy in hydrogen bonding an ionic interactions relative to OPLS4. The simulation boundary was set as a 10 × 10 × 10 Å orthorhombic box. The system was neutralized with the addition of one chloride ion, and the aqueous system was populated with 150 mM sodium chloride to mimic typical biological salt concentrations. All other settings were left on default, and the systems were prepared (∼29k cubic Angstroms and 2600 atoms). To run the simulations of the aqueously buffered PL^Pro^ ligands, the “Molecular Dynamics” function was used with a temperature of 310 K, a simulation time of 100 ns, and recording intervals of 20 ps. The results were visualized in Maestro and analyzed by measuring the dihedral angles of the two rotatable bonds between the piperidinium core and the corresponding benzylic groups (atoms selected for the dihedral measurements starting from the piperidinium core moving toward the benzyl group). Dihedral measurements were plotted for all 5002 frames of the simulations, exported to a.xlsx file from Maestro, and visualized with open-source 2D-dihedral-heatmap software (https://github.com/gentry-lab/2D-dihedral-heatmap).

## Supplementary Material





## Notes

X-ray refinement statistics of compounds, **7** and **41**. X-ray omit and electron density maps of compounds, **7** and **41**. Physicochemical profiling of select cyclic acrylamides (PDF)
Molecular formula strings (CSV)

## Notes

Atom coordinates and structure factors for SARS-CoV-2 PL^Pro^ complexed with ligands can be accessed in the Protein Data Bank via the following accession codes: 9Z0C, compound **7** and 9Z0D, compound **41**. The authors will release the atomic coordinates upon article publication.

## Abbreviations Used

CC50: half maximal cytotoxic concentration
DIPEA: diisopropylethylamine
EtOAc: ethyl acetate
Et_3_SiH: triethylsilane
HATU: hexafluorophosphate azabenzotriazole tetramethyl uronium
HEPES: 4-(2-hydroxyethyl)-1-piperazineethanesulfonic acid
hip: hours post infection
MeCN: acetonitrile
MeOH: methanol
PAMPA: parallel artificial membrane permeability assay
PEG: polyethylene glycol
PL^Pro^: papain like protease
TEA: triethylamine
R_t_: retention time
SARS-CoV-2: severe acute respiratory syndrome coronavirus 2
STAB: sodium triacetoxyborohydride
TRIS: tris­(hydroxymethyl)­aminomethane
