# Supplementary material for: Discovery of Spiro[chromane-2,4′-piperidine] Derivatives as Irreversible Inhibitors of SARS-CoV‑2 Papain-like Protease
Source: J Med Chem. 2026 Feb 2;69(3):3588–608. doi: 10.1021/acs.jmedchem.5c03704 (PMC12910659; doi:10.1021/acs.jmedchem.5c03704)
Supplement: Supplementary file 1 [file jm5c03704_si_001.pdf]

Supplementary Information

Discovery of Spiro[chromane-2,4'-piperidine] derivatives as irreversible inhibitors of SARS-CoV-2 Papain-like Protease

Qiangqiang Wei[†1], Ashley J. Taylor[†2], Nagaraju Miriyala[1], Mahesh A. Barmade[1], Zachary O. Gentry[1], Jordan Anderson-Daniels[2], Kevin B. Teuscher[1], Mackenzie M. Crow[1], Chideraa Apakama[1], Taylor M. South[1], Tyson A. Rietz[1], Kangsa Amporndanai[1], Jason Phan[1], John L. Sensintaffar[1], Mark Denison[2], Taekyu Lee[1], Stephen W. Fesik[*,1,3,4].

†Co-first authors
*Corresponding author

[1] Department of Biochemistry, Vanderbilt University School of Medicine, Nashville, Tennessee, 37232-0146, United States.
[2] Department of Pathology, Microbiology, and Immunology, Vanderbilt University Medical Center, Nashville, TN 37232, USA
[3] Department of Pharmacology, Vanderbilt University School of Medicine, Nashville, Tennessee, 37232-6600, United States.
[4] Department of Chemistry, Vanderbilt University, Nashville, Tennessee, 37235, United States.

Corresponding Author Contact

Stephen W. Fesik
Phone: +1 (615) 322-6303; Fax: +1 (615) 875-3236;
Email: Stephen.fesik@vanderbilt.edu

Table of Contents

X-ray data collection and refinement statistics	S2
Electron density and omit maps for submitted X-ray structures	S3
Physicochemical property profiling of select cyclic acrylamides	S4
Cytotoxicity of covalent inhibitors	S5

X-ray data collection and refinement statistics

Table S1. X-ray data collection and refinement statistics for fragments bound to PL^{Pro}.

	**Compound 7**<br>**9Z0C**	**Compound 41**<br>**9Z0D**
Resolution range	46.24 - 1.9<br>(1.94 - 1.9)	58.59-1.65<br>(1.68 – 1.65)
Space group	P 65 2 2	P 1
Unit cell<br>a, b, c (Å)<br>α, β, γ (⁰)	 77.784, 77.784, 231.185<br>90, 90, 120	 58.050, 63.190, 100.911<br>107.3, 98.0, 98.2
Total reflections	1263207 (60126)	560314 (25991)
Unique reflections	33652 (2068)	155548 (7562)
Multiplicity	37.5 (29.1)	3.6 (3.4)
Completeness (%)	99.9 (98.6)	97.2 (95.3)
Mean I/sigma(I)	31.7 (3.0)	13.4 (1.4)
Wilson B-factor	34.01	26.69
R-merge	0.082 (1.156)	0.035 (0.559)
R-meas	0.084 (1.196)	0.049 (0.791)
R-pim	0.018 (0.302)	0.035 (0.559)
CC1/2	1 (0.0.879)	0.995 (0.691)
Reflections used in refinement	32953 (3195)	155509 (15338)
Reflections used for R-free	1684 (155)	7725 (746)
R-work	0.2081 (0.2961)	0.1996 (0.3087)
R-free	0.2518 (0.3262)	0.2383 (0.3666)
Number of non-hydrogen atoms	0.950 (0.868)	0.957 (0.807)
macromolecules	0.934 (0.808)	0.940 (0.740)
ligands	2549	11285
solvent	2330	9983
Protein residues	30	200
RMS(bonds)	189	1102
RMS(angles)	297	1257
Ramachandran favored (%)	0.007	0.007
Ramachandran allowed (%)	1.01	1.00
Ramachandran outliers (%)	97.25	97.20
Rotamer outliers (%)	2.75	2.80
Clashscore	0.00	0.00
Average B-factor	0.83	1.40
macromolecules	3.53	3.91
ligands	40.20	32.95
solvent	39.95	32.50

Statistics for the highest-resolution shell are shown in parentheses.

Electron density and omit maps of submitted X-ray structures

Table S2. Crystal structures of PL^{Pro} complexed with various ligands (orange sticks) with surface shown in grey and nearby residues as green lines. Composite omit maps: Fo-Fc positive (green mesh) and negative (red mesh) shown at 3.0 σ carved 2.0 Å around the ligand of interest. Electron density maps: 2Fo-Fc (blue mesh) and Fo-Fc positive (green mesh) and negative (red mesh) shown at 1.5, 3.0 σ respectively carved 2.0 Å around the ligand of interest. For compound 41 Cys-111 is also shown as green sticks with its electron density map shown

	Omit map	Electron density map
Compound 7<br>9Z0C	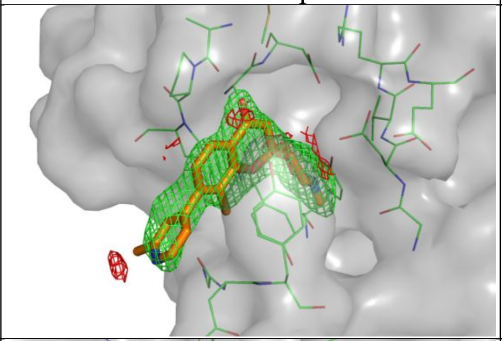	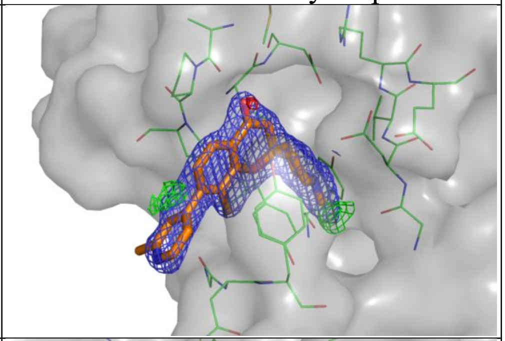
Compound 41<br>9Z0D	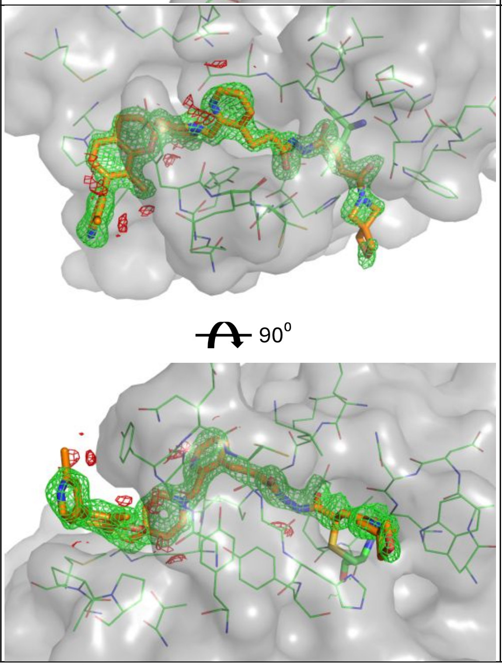	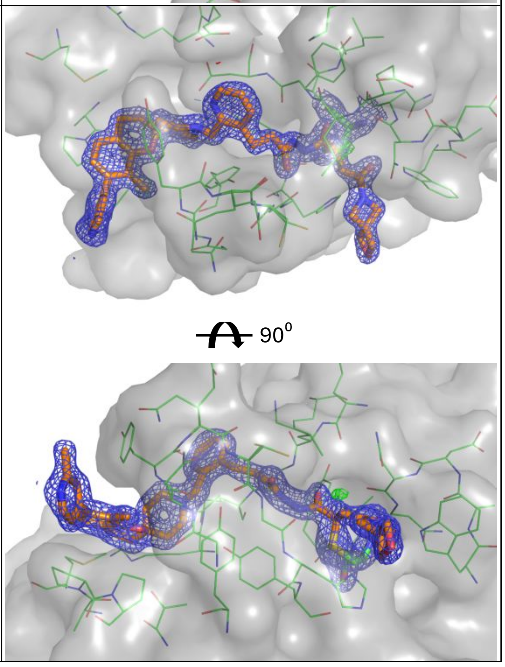

Physicochemical property profiling of cyclic acrylamides

Table S3. Microsome stability and Kinetic solubility of cyclic acrylamides

	Mouse Liver microsome stability					Kinetic solubility (1XPBS pH 7.4)	
Compound	Time Point (min.)	Measured Peak Area	% Remaining	$t_{1/2}$ (min.)	CL_{int} (uL/min/mg protein)	μM	μg/mL
30	0	1063819	100.0%	21.72	63.82	97.5	64.2
	5	823732	77.4%				
	15	595293	56.0%				
	30	330907	31.1%				
	60	154090	14.5%				
33	0	174036	100.0%	63.60	21.79	137.9	91.8
	5	161840	93.0%				
	15	157416	90.5%				
	30	128434	73.8%				
	60	90388	51.9%				
34	0	901622	100.0%	70.64	19.62	138.6	92.4
	5	782845	86.8%				
	15	728118	80.8%				
	30	637302	70.7%				
	60	479677	53.2%				

Table S4. Pampa permeability of cyclic acrylamides

	Pampa permeability						
Compound	ACCEPTOR	DONOR	C0	Ceq	Pe	%R	logPapp
30	< 0.4	113.8	75.9	68.44	1.30E-07	-0.50	-6.89
33	2.1	151.9	148.5	91.98	5.13E-07	-0.03	-6.29
34	< 0.4	145.7	137.8	87.58	1.02E-07	-0.06	-6.99

Physicochemical property profiling of cyclic acrylamides

Table S5. Cytotoxicity of tested compounds

Compound	A549 EC_{50}	A549 CC_{50}
**33**	>100	>100
**34**	20	>100
**35**	>100	>100
**36**	>100	>100
**37**	>100	>100
**38**	>100	>100
**39**	8.6	>100
**40**	12.6	>100
**41**	27.9	>100

Compound	A549 EC_{50}	A549 CC_{50}
**42**	8.5	>100
**43**	81.0	>100
**44**	17.1	>100
**45**	2.1	>100
**46**	13.7	>100
**47**	19.2	>100
**48**	10.1	>100
**49**	13.8	>100
**50**	102	>100
